# Host adaptation drives genetic diversity in a vector-borne disease system

**DOI:** 10.1093/pnasnexus/pgad234

**Published:** 2023-08-08

**Authors:** Matthew A Combs, Danielle M Tufts, Ben Adams, Yi-Pin Lin, Sergios-Orestis Kolokotronis, Maria A Diuk-Wasser

**Affiliations:** Department of Ecology, Evolution and Environmental Biology, Columbia University, New York, NY 10027, USA; Department of Epidemiology and Biostatistics, School of Public Health, SUNY Downstate Health Sciences University, Brooklyn, NY 11203-2098, USA; Institute for Genomics in Health, SUNY Downstate Health Sciences University, Brooklyn, NY 11203-2098, USA; Department of Ecology, Evolution and Environmental Biology, Columbia University, New York, NY 10027, USA; Infectious Diseases and Microbiology Department, University of Pittsburgh, Pittsburgh, PA 15261, USA; Department of Mathematical Sciences, University of Bath, Bath, BA27AY, UK; Division of Infectious Diseases, Wadsworth Center, New York State Department of Health, Albany, NY 12201, USA; Department of Biomedical Sciences, University at Albany, Albany, NY 12203, USA; Department of Epidemiology and Biostatistics, School of Public Health, SUNY Downstate Health Sciences University, Brooklyn, NY 11203-2098, USA; Institute for Genomics in Health, SUNY Downstate Health Sciences University, Brooklyn, NY 11203-2098, USA; Division of Infectious Diseases, Department of Medicine, College of Medicine, SUNY Downstate Health Sciences University, Brooklyn, NY 11203-2098, USA; Department of Cell Biology, College of Medicine, SUNY Downstate Health Sciences University, Brooklyn, NY 11203-2098, USA; Department of Ecology, Evolution and Environmental Biology, Columbia University, New York, NY 10027, USA

**Keywords:** zoonosis, *Borrelia*, host, vector, pathogen, adaptation, multiple niche polymorphism, frequency-dependent selection

## Abstract

The range of hosts a pathogen can infect is a key trait, influencing human disease risk and reservoir host infection dynamics. *Borrelia burgdorferi* sensu stricto (*Bb*), an emerging zoonotic pathogen, causes Lyme disease and is widely considered a host generalist, commonly infecting mammals and birds. Yet the extent of intraspecific variation in *Bb* host breadth, its role in determining host competence, and potential implications for human infection remain unclear. We conducted a long-term study of *Bb* diversity, defined by the polymorphic *ospC* locus, across white-footed mice, passerine birds, and tick vectors, leveraging long-read amplicon sequencing. Our results reveal strong variation in host breadth across *Bb* genotypes, exposing a spectrum of genotype-specific host-adapted phenotypes. We found support for multiple niche polymorphism, maintaining *Bb* diversity in nature and little evidence of temporal shifts in genotype dominance, as would be expected under negative frequency-dependent selection. Passerine birds support the circulation of several human-invasive strains (HISs) in the local tick population and harbor greater *Bb* genotypic diversity compared with white-footed mice. Mouse-adapted *Bb* genotypes exhibited longer persistence in individual mice compared with nonadapted genotypes. Genotype communities infecting individual mice preferentially became dominated by mouse-adapted genotypes over time. We posit that intraspecific variation in *Bb* host breadth and adaptation helps maintain overall species fitness in response to transmission by a generalist vector.

**Significance Statement**

Lyme disease is the most common vector-borne disease in the United States, with a causative agent (*Borrelia burgdorferi*, Bb) exhibiting high genetic diversity that partially correlates with human disease manifestations. Understanding the extent of host adaptation in pathogens is critical for evaluating disease risk, but host specificity and mechanisms maintaining genetic diversity in Bb are unknown. We show that Bb genotypes exhibit variable host adaptation to white-footed mice and passerine birds, two common reservoir hosts, which appears to promote high intraspecific pathogen diversity. Conversely, we find limited evidence of negative frequency-dependent selection, an alternative mechanism for diversity maintenance. Our results reveal intraspecies host breadth variation and suggest that host competence for *B. burgdorferi* sensu stricto is a pathogen genotype-specific trait.

## Introduction

Evaluating human disease risk from zoonotic pathogens, those shared between wildlife and humans, requires the characterization of pathogen traits influencing their environmental distribution and spillover potential (1). Recent meta-analyses and reviews have repeatedly identified host breadth, or the capacity to infect phylogenetically diverse species, as a key trait influencing spillover (2–4). Greater host breadth provides pathogens with more opportunities for population persistence across heterogeneous environments (e.g. variable host availability), but often invokes a fitness tradeoff as pathogens must adapt to diverse immunological selection pressures (5, 6).

Wider pathogen host breadth is intrinsically linked to increased competence of potential hosts, defined as the capacity to acquire, maintain, and transmit infection (7). Though host competence is increasingly used to model human disease risk, unexplained sources of variation can limit its utility (7–9). In particular, intraspecific genetic variation in multistrain pathogens may influence host–pathogen interactions, preventing accurate characterization of pathogen host breadth and host competence (10, 11).

To understand the consequences of genotypic diversity on pathogen phenotypes and human disease risk, one must also characterize the evolutionary drivers that maintain diversity and the ecological context in which variation is observed. Balancing selection can maintain diverse genotypes across a bacterial population when heterogeneous immunological and physiological host environments select for different traits (5, 12, 13). While observing evidence of balancing selection can be straightforward (e.g. by observing genotypic frequencies), identifying the eco-evolutionary processes responsible for these patterns requires careful experimental design or intensive and targeted population sampling (12, 14–17). Thus, to evaluate diversity patterns and evolutionary drivers of pathogen traits, it is crucial to sample pathogens across endemic hosts and time scales relevant to detect natural selection (18–21).

Vector-borne pathogens are increasingly responsible for emerging infectious diseases (22, 23) and may provide unique insight into the links between the evolution of host breadth and the ecology of human disease risk. *Borrelia burgdorferi* sensu stricto (hereafter *Bb*) is a spirochete bacterium that causes 476,000 human cases of Lyme disease in the United States annually (24). Lyme disease is the most common vector-borne disease in the United States and continues to increase in geographic range and case numbers (25, 26), particularly in the Northeast and Midwest United States, where *Bb* circulates primarily in small mammal and bird species via the generalist tick vector *Ixodes scapularis* (27). Despite the increasing toll of tick-borne pathogens on human health, fundamental questions about their basic biological traits, including host breadth and the role of immune environments in structuring underlying selection mechanisms, remain unanswered (11, 28, 29).

Genetic studies of *Bb* regularly identify elevated genetic diversity and signatures of balancing selection at the pathogen's outer surface protein C (*ospC*) locus in natural populations of infected ticks, with more than 25 known alleles (30–33). The OspC protein is required for host infection (34) and *ospC* alleles are often used to distinguish among strains with variable phenotypes, including a subset deemed human invasive strains (HISs) that exhibit more severe pathology in humans (35–37). The eco-evolutionary drivers maintaining *ospC* variation and its implications for human disease risk remain a focus of active debate (38). Two competing hypotheses exist, centered around distinct host–pathogen interactions. The first suggests *Bb* diversity is maintained via multiple niche polymorphism (MNP), in which genotypes exhibiting host-adapted phenotypes segregate across different host species (39, 40). The strength of selective pressure can vary across hosts and time in a context-dependent fashion, thus shaping an evolving adaptive landscape. The second predicts that negative frequency-dependent selection (NFDS) occurs via host antibodies, which iteratively induce fitness costs on common genotypes within the local population, driving temporal fluctuations in genotype frequency (41, 42).

Here, we present the most comprehensive long-term study of *Bb ospC* diversity to date examined across two divergent reservoir host types, passerine birds and white-footed mice, as well as tick vectors in the United States. By leveraging long-read amplicon sequencing of the *ospC* locus, we reveal intraspecific variation in host-adaptation phenotypes that yield support to the MNP hypothesis, while the absence of strain dominance shifts indicates a limited role for NFDS in maintaining strain diversity. Our results shed light on the drivers of balancing selection, the evolution of host breadth, and their implications for human disease risk for this emerging zoonotic pathogen. Further, our results suggest the presence of unique infectivity profiles for genotypes of *Bb*, indicating that host competence is a *Bb* genotype-specific trait that modifies the contribution of host species to overall *Bb* infection.

## Results

### Genotypic community structure

To characterize the distribution and diversity of *ospC* major groups (hereafter referred to as genotypes), we sequenced the entire *ospC* locus from 553 white-footed mice, 92 passerine birds (11 species, Table S1), and 628 individual nymphal *I. scapularis* ticks. In total, we assigned 696,453 HiFi reads to 21 genotypes (Supplementary File S1), while sequencing depth per sample varied between 20 and 9,874 reads. For every sample, the genotypes of *Bb* infections were characterized by independently matching each read with a single *ospC* cluster. At least one genotype was present in each sample, and infection with multiple genotypes (hereafter, coinfection) was common across all samples (52.8%). Genotype richness ranged from 1 to 16 within individual samples, and was lower on average in mice (*α* = 1.77) than in birds (*α* = 2.59) and intermediate in nymphal ticks (*α* = 2.30). Sequencing depth was correlated with genotype richness only at shallower coverage below 100 reads (*P* < 0.001; Fig. S1). *Bb* diversity and evenness in mice were lower compared with those of birds and nymphs, despite similar richness across populations (Fig. S2).

We identified two novel *Bb* genotypes. One was designated subtype Cj and shares 97.3% sequence identity with *Bb* type C, but contains a 75-bp region that is fully identical with *Bb* type J—evidence of a recent recombination event (see below). Another genotype exhibited >8% sequence dissimilarity with all known genotypes and was found almost exclusively in birds (present in 0.2% of *Bb*-infected mice compared with 17.4% of *Bb-*infected birds), which we designated type J3. We also identified a genotype that likely represents infection by *Borrelia kurtenbachii*, a recently described genospecies and close relative of *Bb* that infects mammals exclusively (43). The *Borrelia kurtenbachii* genotype detected (*B.kurt*) matched a known *Borrelia kurtenbachii ospC* sequence with 0.02% dissimilarity, while its closest match to a known *Bb* genotype (type T) was at 11.2% dissimilarity. *B.kurt.* was the only genotype never identified in birds (Table S1) and exhibited a distinct lack of co-occurrence with other genotypes in mice (Fig. 3), indicating a low frequency of mixed infection between *Bb* and *B.kurt.* among mice (4.8% of *Bb*-infected mice).

### Evidence supporting MNP mechanisms in hosts

**Fig. 1.**
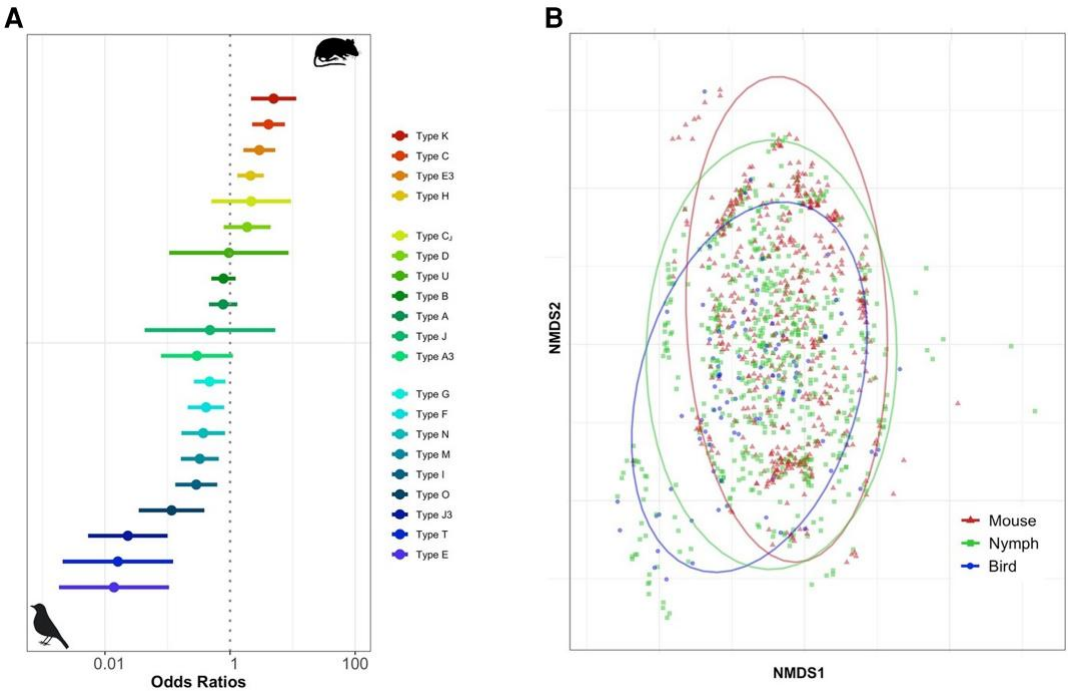
Host associations among *ospC* genotypes. A) Log odds of infection with each genotype in mice compared with bird hosts. Significant associations exhibit confidence intervals that do not overlap the dotted line. B) NMDS plot of individual mouse, nymph, and bird genotype communities with 95% confidence intervals.

If genotypic diversity in *Bb* communities is maintained by MNP, we expect significant associations between genotypes and hosts, with nymphal ticks harboring all circulating genotypes. We identified strong patterns of host association among the 20 *Bb* genotypes found in this study (Fig. 1A, Table 1). Four genotypes (types C, E3, H, and K) were significantly more likely to be found in rodents compared with avian hosts, whereas nine genotypes (types E, F, G, I, M, N, O, T, and CJ) followed the opposite taxonomic trend. The remaining seven genotypes (types A, A3, B, D, J, U, and J3) were not associated with either host taxon. We hereafter refer to these genotypes as mouse adapted, bird adapted, and generalist, respectively. The signal of host association among the most strongly bird-adapted genotype (type E), which had merely a 1.0% probability of infecting a mouse (odds ratio [OR] = 0.01), is stronger than that of the most mouse-adapted genotype (type K), which maintains a 16% probability of infecting a bird (OR = 0.20, when mouse is set as the reference). No *Bb* genotypes were found exclusively in a single host taxon, indicating that even mouse-adapted genotypes can infect birds, and vice versa (Table S1). In the majority of hosts, the dominant genotype, accounting for the largest proportion of reads, was adapted to that host taxon. This occurred in 376 of 553 individual mice (68.0%) and 51 or 92 individual birds (55.4%).

**Table 1.** Log odds ratio of strain-specific infection in mouse hosts and tick vectors relative to bird hosts.

	Mouse		Tick	
*ospC* genotype	Odds ratio	*P*-value	Odds ratio	*P*-value
K	**4.97** (**2.16–11.43)**	<0.001	**2.32** (**1.00–5.37)**	0.050
C	**4.12** (**2.25–7.53)**	<0.001	**2.74** (**1.50–4.99)**	0.001
E3	**2.94** (**1.63–5.28)**	<0.001	**2.31** (**1.29–4.14)**	0.005
H	**2.13** (**1.31–3.46)**	0.002	1.10 (0.67–1.80)	0.705
CJ	2.17 (0.50–9.44)	0.300	1.98 (0.47–8.45)	0.354
D	1.88 (0.79–4.44)	0.153	2.02 (0.87–4.70)	0.103
U	0.96 (0.11–8.60)	0.968	0.16 (0.06–0.43)	<0.001
B	0.78 (0.50–1.23)	0.285	0.84 (0.55–1.29)	0.423
A	0.77 (0.46–1.31)	0.338	0.70 (0.42–1.17)	0.173
J	0.48 (0.04–5.28)	0.546	2.47 (0.33–18.82)	0.382
G	**0.47** (**0.26–0.84)**	0.011	0.84 (0.50–1.41)	0.505
F	**0.41** (**0.21–0.81)**	0.010	1.12 (0.63–2.00)	0.700
N	**0.37** (**0.17–0.83)**	0.016	1.24 (0.63–2.44)	0.529
M	**0.33** (**0.16–0.66)**	0.002	0.88 (0.49–1.59)	0.680
A3	**0.30** (**0.08–1.11)**	0.071	0.41 (0.13–1.30)	0.130
I	**0.29** (**0.13–0.62)**	0.002	**0.45** (**0.23–0.89)**	0.021
O	**0.12** (**0.03–0.39)**	<0.001	**0.16** (**0.06–0.43)**	<0.001
J3	**0.02** (**0.01–0.10)**	<0.001	**0.32** (**0.18–0.56)**	<0.001
T	**0.02** (**0.00–0.12)**	<0.001	**0.16** (**0.06–0.43)**	<0.001
E	**0.01** (**0.00–0.11)**	<0.001	**0.21** (**0.11–0.41)**	<0.001

Significant odds ratios are in bold and 95% confidence intervals provided in parentheses.

We also represented individual hosts and ticks by the community of detected *Bb* genotypes, using sequencing depth as a proxy for ranking the relative abundance within individual infections. To characterize the similarity among individual genotypic communities among the populations of mice, birds, and ticks, we used nonmetric multidimensional scaling (NMDS). NMDS revealed moderate separation between genotypic communities found in each sampled population, with birds and mice clustering further away from one another, and ticks forming an intermediate cluster with community characteristics that overlap with both host groups (Fig. 1B). To quantify the degree of distinctiveness among host and tick communities, we used a pairwise similarity test of genotype overlap among populations, and found that both mammal and bird genotype communities were more similar to nymph genotype communities (0.904 and 0.883, respectively), than they were to each other (0.672; Table S2). We observed some variations in the frequency of genotype infection across *Bb-*infected bird species (Table S3), but generalized linear models (GLMs) differentiating between the three most commonly infected bird species (Carolina wren, common yellowthroat, and American robin) indicated that most genotypes exhibited parallel responses to individual *Bb* genotypes compared with white-footed mice (Fig. S3). We thus combined bird-derived data in our analyses.

### Limited evidence of NFDS mechanisms in hosts

**Fig. 2.**
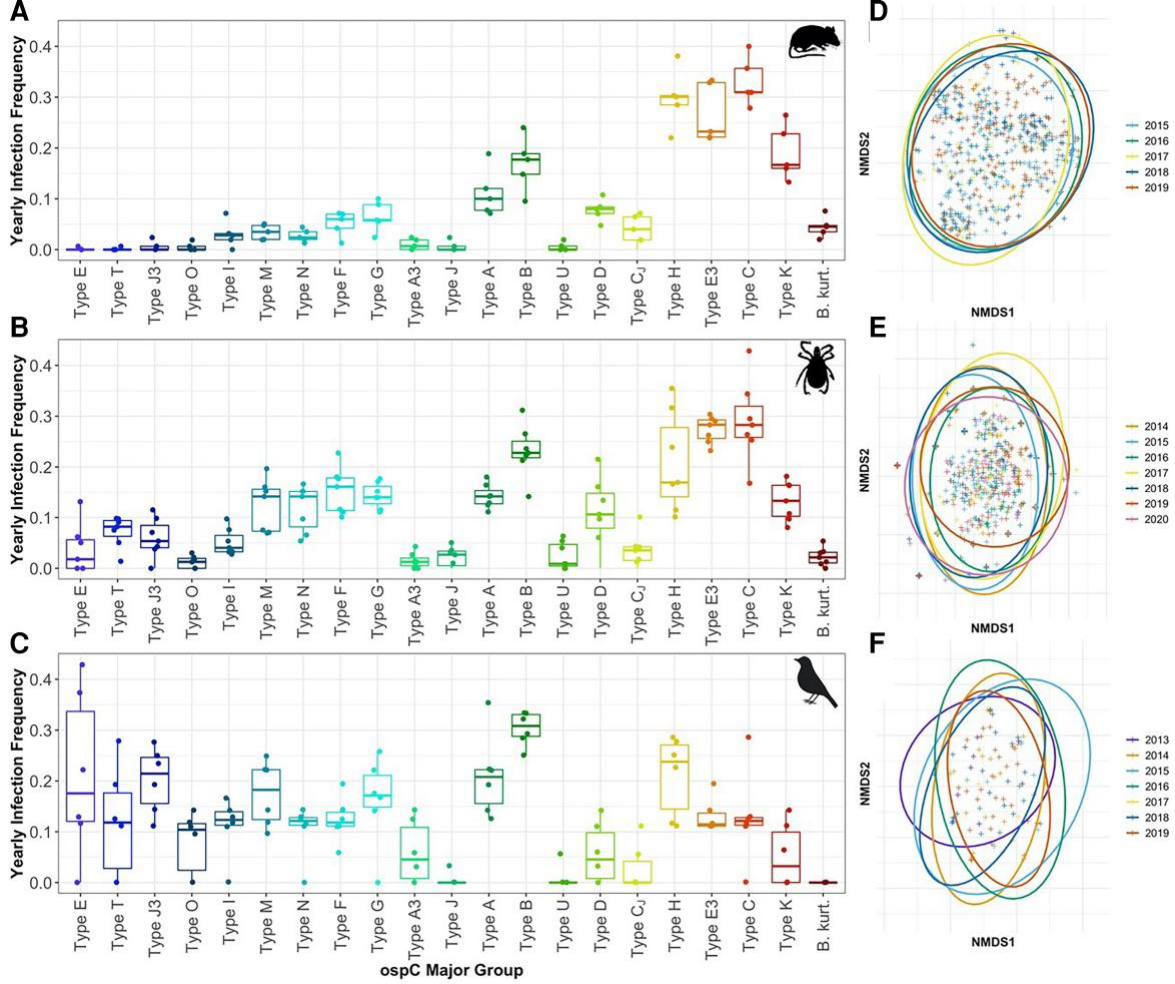
Yearly trends in *ospC* genotype frequency and individual host *Bb* community structure. Boxplots of *ospC* genotype frequency distributions observed in A) mice, B) nymphs, and C) birds. NMDS plots of individual *Bb* communities separated by year among D) mice, E) nymphs, and F) birds with 95% confidence ellipses. Boxplot colors reflect the strength of the host association shown in Fig. 1.

If genotypic diversity in *Bb* is maintained by NFDS, we would expect significant variation in the dominant genotype and shifting genotypic frequencies within host populations over time. We first examined the genotype frequency and similarity of individual genotype communities within populations across years (Fig. 2). For mice, where sample sizes among years were consistently high, the distributions of annual genotype frequencies exhibited low dispersion over time (Figs. 2A and S4). Similar patterns were observed among nymphal ticks and bird hosts, though smaller sample sizes and variation among bird species led to more variation in frequency distributions (Figs. 2A, S5, and S6).

We used analysis of similarities (ANOSIM) to test for significant differences in the composition of individual communities within and between years for each taxonomic population, separating analyses across sampling sites for mice and nymphal ticks. We found significant differences in mouse genotype communities across years at only one of the three sampling sites (Rodman's Hollow [RH]: *P* = 0.047, Block Island National Wildlife Refuge [NR]: *P* = 0.09, private property on eastern part of the island [EI]: *P* = 0.673), but not among bird infection communities, which were sampled at multiple sites across the island (*P* = 0.85). Pairwise post-hoc comparisons revealed significant differences in mice at RH only between 2015 and 2016, 2018, and 2019. In contrast, questing nymphs exhibited significant yearly differences in their infection community across all three sites (RH: *P* = 0.001, NR: *P* = 0.001, EI: *P* = 0.004). Graphical examination using NMDS plots revealed strong and consistent overlap for within-year variation in genotype communities across populations of mice, birds, and ticks, indicating that communities remain similar between years (Fig. 2D-F).

### Divergent genotype co-occurrence patterns among hosts

**Fig. 3.**
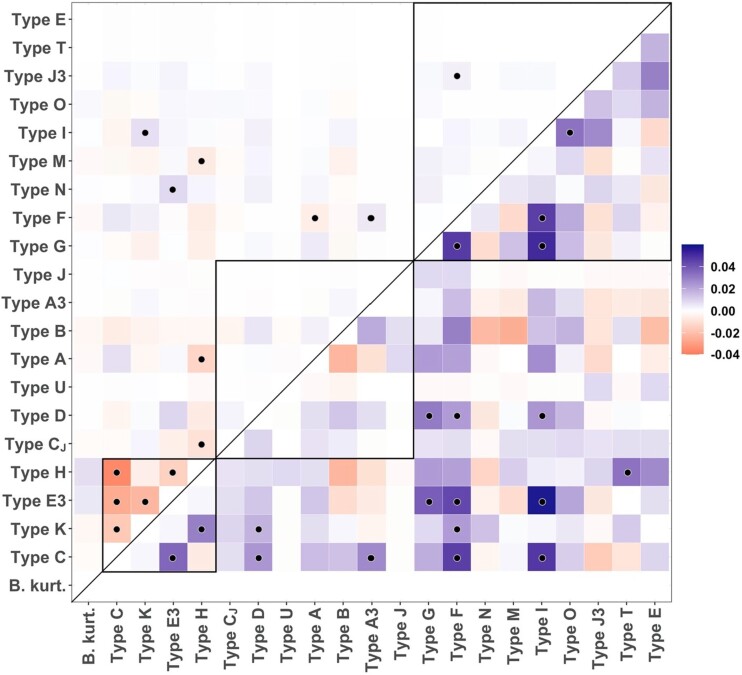
Heatmap of effect size for co-occurrence probability estimates for *ospC* genotypes found in individual mammal (top triangle) or bird hosts (bottom triangle). Color indicates the strength of the effect (blue = positive, red = negative), and black dots indicate significant associations (*P* < 0.05). Black boxes within the heatmap surround the putative mouse-adapted genotypes (bottom), generalist genotypes (middle), and bird-adapted genotypes (top).

Competitive or facilitative interactions among genotypes within hosts may influence their probability of co-occurrence within the same individual host community. For each population of mice, birds, and ticks, we compared the observed pairwise co-occurrence of genotypes within individual infection communities to the random distributions predicted by the total number of genotype occurrences in that respective population. We found contrasting patterns in the number and directionality of significant genotype pairs across mice and birds (Fig. 3). Among mice, we observed 13 significant pairwise correlations, including multiple negative correlations among the four mouse-adapted genotypes (i.e. pairs of genotypes were observed together less frequently than expected). Among birds, we observed a greater total number of significant correlations (*n* = 19) than in mice, all of which were positive (i.e. pairs of genotypes were observed together more frequently than expected), with no consistent relationship among bird-adapted genotypes. Among nymphs, we observed both positive and negative significant correlations among genotypes, with strong negative interactions between those genotypes with the strongest signals of adaptation to birds and mice, suggesting ticks do not often acquire both mouse- and bird-adapted genotypes through a single larval blood meal (Fig. S7).

We also asked whether evolutionary differences among genotype pairs, specifically genetic dissimilarity, and phylogenetic network distance, explained the strength of observed co-occurrence relationships using linear models. We found no significant relationship either between predictor and co-occurrence in mice (*P* = 0.097 and *P* = 0.096) or nymphs (*P* = 0.087 and *P* = 0.082), but a significant negative relationship between predictors and co-occurrence was found in birds (*P* = 0.007 and *P* = 0.008; Fig. S8), indicating that co-occurrence was less frequent when genotype pairs were more genetically divergent.

### Individual mouse genotype community dynamics

The genotype community of individual hosts may change over time due to the host's ability to clear infections by specific genotypes and due to sequential introductions through multiple nymphal tick bites. To understand how patterns of genotype host adaptation influence individual-level infection dynamics, we used a multistate Markov (MSM) model to dissect mouse genotype infection in mice sampled multiple times within a single year. We characterized the state of infection based on the identity of the dominant genotype (i.e. greatest sequencing depth) at each sampling time, specifying three possible states: (i) uninfected, (ii) infected with a mouse-adapted genotype, (iii) infected with a nonmouse-adapted genotype (i.e. generalist or bird adapted). We found that mice in any initial state are more likely to transition to a mouse-adapted genotype infection than a nonmouse-adapted genotype infection (Table 2). We also found that the mean persistence (i.e. mean sojourn time) of infections was nearly three times greater in mouse-adapted genotypes than that in nonmouse-adapted genotypes in mice (27.6 vs. 9.5 days; Table S4). Yet, when the state of an uninfected mouse changed, we found it was twice as likely to become infected with a nonmouse-adapted genotype than a mouse-adapted genotype (Table S5). The dominant genotype was significantly more likely to change with increasing time since initial capture (Fig. S9), but was not influenced by individual characteristics or tick burden (Table S6). Together, our model suggests that while infections with nonmouse-adapted genotypes are common in mice, these genotypes exhibit weak persistence and are often replaced with infections dominated by mouse-adapted genotypes.

**Table 2.** Transition probabilities between infection states in white-footed mice estimated over a 4-week period with the MSM model.

	Uninfected	Adapted	Nonadapted
Uninfected	0.722 (0.664–0.765)	0.346 (0.277–0.258)	0.167 (0.121–0.237)
Adapted	0.164 (0.131–0.212)	0.501 (0.395–0.580)	0.336 (0.252–0.42.7)
Nonadapted	0.114 (0.088–0.146)	0.152 (0.111–0.205)	0.497 (0.401–0.592)

Columns and rows represent starting and transition states, respectively. 95% Confidence intervals are provided in parentheses.

### Evolutionary patterns among genotypes

**Fig. 4.**
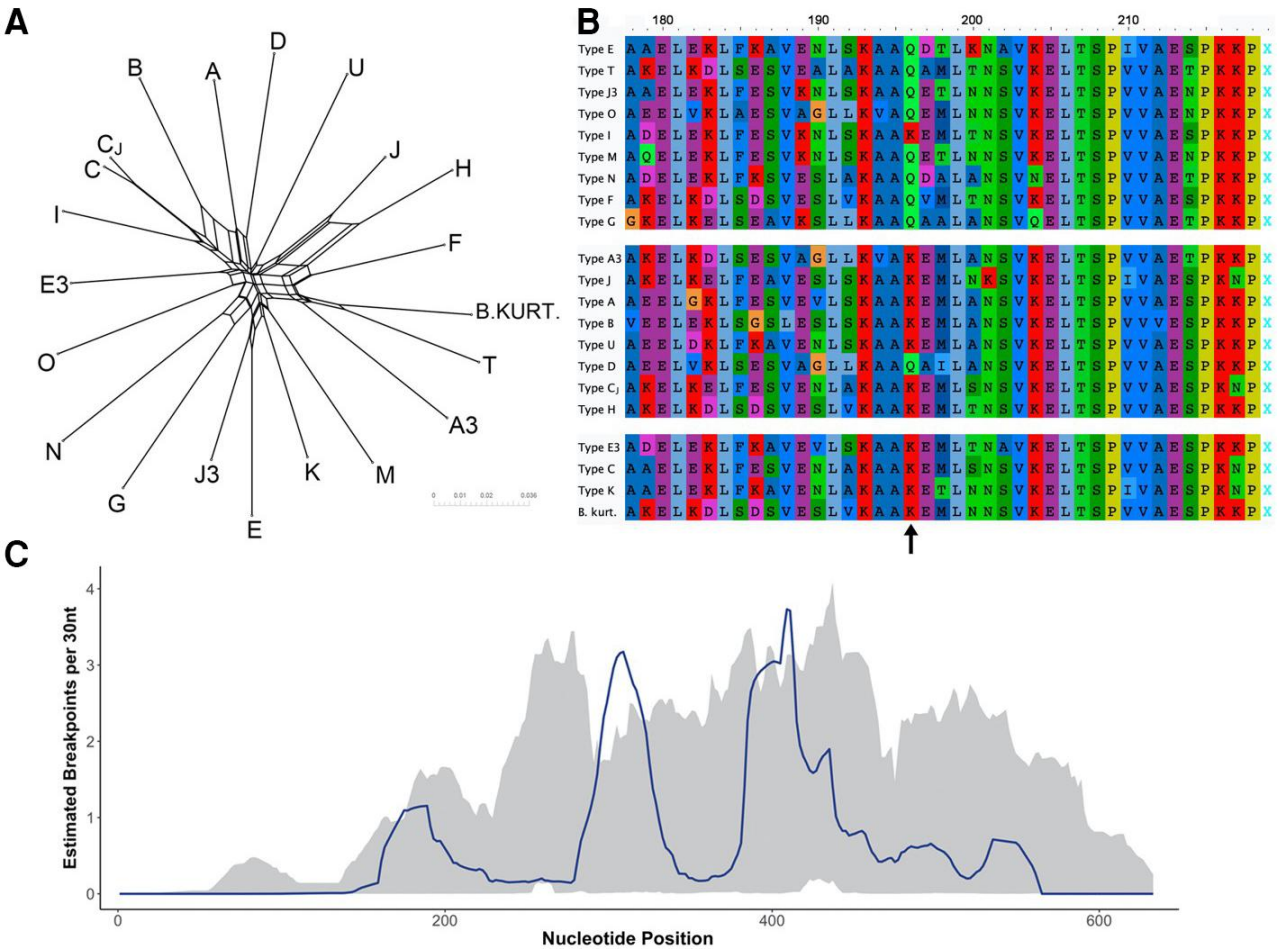
Evolutionary patterns at the *ospC* locus. A) Phylogenetic network of 21 observed genotypes. B) Amino acid alignment of the 5′ end of *ospC* gene with an arrow identifying the K196Q mutation. Genotypes are separated by phenotypic groups: bird-adapted (top), generalist (middle), mouse-adapted (bottom). C) Recombination breakpoint estimation (dark line) across the *ospC* locus. Shaded area represents 95% confidence intervals for the local hot/cold spot test. Areas where observed counts exceed the confidence interval are inferred to be recombination hot spots.

The Neighbor-Net phylogenetic network revealed long branches separating each *ospC* type, with pronounced reticulation at the center of the network (Fig. 4A). The single exception was between type C and type J3, which are closely related (97.3% nucleotide similarity). Examination of genotype-representative amino acid sequences revealed a K196Q mutation, located at the C-terminus of OspC's 5th α-helix. All mouse-adapted genotypes and all but one of the generalist genotypes exhibited a lysine at this residue, while all but one bird-adapted genotypes exhibit a glutamine (Fig. 4B).

Using a suite of recombination detection tools, we identified 11 intralocus recombination events across the 21 *ospC* types, each of which was detected by at least three independent methods (Table S7). Analysis of breakpoints suggested two major recombination hot spots around nucleotide positions 300 and 400, with lower confidence hot spots in positions 180 and 500–550 regions (Fig. 4C).

## Discussion

A critical step in evaluating human disease risk from emerging zoonotic pathogens is understanding how interactions with endemic reservoir hosts shape a pathogen's genetic and functional diversity, in particular its host breadth and ability to infect new species (2, 44). Here, we demonstrate that different genotypes of *Bb*, a generalist vector-borne zoonotic pathogen, exhibit strong differentiation in their capacity to infect reservoir host species associated with *ospC* alleles. Together, the 20 observed genotypes exhibited a full spectrum of host-adapted phenotypes across white-footed mice and passerine birds. These results reveal intraspecific variation in pathogen host breadth and the strength of host-specific adaptation. This intraspecific variation may represent an evolutionary strategy by which *Bb* maintains overall species fitness in response to transmission by a generalist vector.

The evolutionary drivers maintaining *ospC* polymorphisms in nature have been debated in recent decades. Some field-based studies have suggested MNP drives *Bb* diversity through evidence of genotype–host associations defined by genotypes (39, 45) or multilocus sequence typing (40), though others found no such pattern (46). Laboratory-based studies have observed variable infection establishment, persistence, and transmission of *Bb* genotypes within and among host species (47–51). Yet, other short-term field studies (i.e. up to 3 years) have found shifting frequencies of *Bb* genotypes in tick vectors consistent with balancing selection mediated by NFDS and mixed evidence supporting MNP with little to no interannual frequency variation (30, 31, 52). Pervasive local recombination, revealed by recent *Bb* comparative genomics, appears to drive genetic and antigenic diversity at *ospC*, and NFDS is proposed as a parsimonious explanation for the maintenance of this variation, particularly if recombination disrupts host–pathogen coadaptation (29, 41, 53). Our results, spanning multiple host taxonomic groups and nymphal ticks over up to 7 years, provide strong evidence supporting MNP in structuring *Bb* genetic diversity. While we observed little evidence of yearly temporal shifts in population-level genotype frequency, as predicted by NFDS, it is possible that antigenic exclusion in individual hosts accounts for minor variation among host-adapted genotypes. Mice on Block Island are an isolated population and likely exhibit decreased genetic diversity. The impact of reduced host diversity on adaptation by *Bb* genotypes, and the consistency to which host-adapted genotypes are also adapted to the same host species with different genetic backgrounds is unclear and presents an avenue for further investigation.

While MNP appears to partially maintain *Bb* diversity through genotype-specific host adaptation, variable OspC proteins are also clearly under selection for distinct antigenic epitopes that limit cross-reactivity (54). Since processes governing initial establishment of infection occur before the production of antibodies, additional selective forces must maintain diverse antigenic epitopes (55). We propose that one mechanism maintaining *ospC* diversity is limited antibody cross-reactivity facilitates host adaptation through MNP by enabling serial susceptibility (i.e. superinfection) of hosts. Because hosts produce antibodies with increased specificity targeting the epitope of the infecting type (56, 57), subsequent infections by different genotypes encounter essentially naïve hosts. Thus, short-lived infections by nonadapted genotypes should not restrict the future success of more host-adapted genotypes, improving the likelihood of transmission for the species despite variable infection success by individual genotypes. In the Northeastern United States, nymphal ticks are abundant for 6–8 weeks, several weeks before the emergence of larvae, allowing hosts to experience multiple *Bb* introductions from independent tick bites. Longer duration phenotypes then have a higher likelihood of infecting the next tick cohort's larvae (58, 59). Indeed, synchronous nymph-larval tick phenology has been shown to be associated with infection persistence (60). In our study, not only did mouse-adapted genotypes exhibit much longer persistence times in mice sampled multiple times than nonmouse-adapted genotypes, but mouse infection communities preferentially shifted to mouse-adapted genotype dominance over time. While temporal shifts in *Bb* infections have previously been observed in mice and humans (61–63), our results highlight how the combination of limited antibody cross-reactivity and variable host-adapted pathogen genotypes may provide a fitness advantage for *Bb* when hosts experience multiple independent infections.

A second mechanism maintaining genotype diversity is the lack of complete host specialization. Even *Bb* genotypes with the strongest evidence for host adaptation were observed in their nonadapted hosts. This pattern may be due to intraspecific variation in host competence or result from facilitative interactions among genotypes that allow nonadapted genotypes to persist in the presence of host-adapted genotypes (7, 64). Experimental studies of bacterial evolution have shown that increasing environmental complexity (e.g. host diversity) results in overlapping niches and imperfect specialization (65). A recent model examining the interaction between MNP and NFDS in *Bb* also suggested that more diverse suites of differentially host-adapted phenotypes can coexist in a population when antigenic variation, a form of environmental heterogeneity, is high (66). These findings are suggestive of a role for host-adaptation-related pathogen traits in maintaining pathogen fitness and persistence in infected hosts. The high genotype diversity observed in *Bb* appears to fit a “mass effect” metacommunity model (67, 68), where host-adapted genotypes are common in their respective hosts and strong propagule pressure (from infectious ticks) results in a low frequency of nonadapted genotypes co-occurring with adapted ones.

### Host community composition drives patterns of human disease risk

Host competence for vector-borne pathogens is defined as the capacity of hosts to permit the establishment, development, and transmission of an infection (69). Because all hosts in the community are exposed to the same frequency of genotypes locally present in host-seeking nymphs (Fig. 2B), observed differences among hosts in pathogen genotypes (Fig. 2A and C) reflect differences in at least some components of host competence. These field results are consistent with our previous transmission experiments, which assessed all components of reservoir competence as well as cellular and molecular mechanisms (49) and mathematical models demonstrating the potential role of MNP in maintaining OspC diversity (66). We thus propose that the host competence trait (70) should be expanded to consider the infecting *Bb* genotype that modifies the contribution of host species to overall *Bb* infection.

Of particular interest is the role of reservoir hosts in transmitting HISs. We found that the frequency of specific *Bb* genotypes in nymphal ticks matches the genotype prevalence observed in the local host community (Fig. 2A). This trend is particularly important for understanding the role of the host community composition on the local prevalence of HISs, a subset of *Bb* genotypes, including *ospC* types A, B, I, K, and N that produce more severe human disease outcomes (36, 71, 72). Our results suggest that human disease risk for HIS relies in part on the specific suite of local reservoir hosts and the strength of their associations with different HIS genotypes. Past research has sought to link human disease risk for *Bb* with the presence of amplification or dilution reservoir hosts, those exhibiting higher or lower host competence, respectively (70, 73). Our results indicate that “low-competence” hosts can still be responsible for the preferential transmission of human-invasive genotypes, thus amplifying disease risk.

In our study, birds were the main source of *ospC* types I and N, and harbored types A and B in high frequency, although not as high as mice do. Though *Bb* infection prevalence varies among bird species (18, 74), previous work estimated that 27% of larval ticks feed on birds at our study location (75). We found higher genotype richness and evenness in bird hosts compared with white-footed mice. Further, individual bird infection communities were characterized by greater genotype co-occurrence, indicating facilitation, in contrast with mammal communities that appear to exhibit only competitive interactions among genotypes. These findings may be partially explained by birds' longer lifespans, different immunity mechanisms, larger home ranges, or other life-history traits resulting in more opportunities for infection (76). Thus, our study emphasizes the role of passerine birds in maintaining *Bb* transmission and increasing human disease risk locally, in addition to their known roles as reservoir hosts and tick dispersers (18, 45, 74, 77–79).

### Signatures of adaptation in *ospC*

*Bb* genomes are characterized by the presence of paralogous lipoprotein gene families, whose members provide multiple and redundant roles throughout different stages of infection (80, 81), though o*spC* has no close paralogs (82). The OspC protein has known roles binding to multiple host ligands to promote evasion of host complement, binding to tick salivary proteins to prevent host antibody attack, and promoting host tissue colonization and dissemination (37, 83–86). The *ospC* K196Q mutation we identified may contribute to host-adaptation *Bb* phenotypes, as it was observed in eight out of nine bird-associated genotypes, but only in one out of seven generalist genotypes, and was absent in mouse-adapted genotypes. This mutation localizes in the N-terminus of OspC's 5th α-helix, a region predicted to be important for genotype-specific antibody cross-reactivity (54). Though *ospC* variation clearly impacts antigenic recognition, the elevated role of MNP identified here suggests it may also influence host-specific adaptation. If this gene is involved in host-specific complement evasion or tissue dissemination in addition to influencing antibody cross-reactivity, it would be suggestive of competing or complementary evolutionary pressures governing standing variation at the *ospC* locus. Importantly, we posit that genotype identity alone does not dictate host-adaptation phenotypes, as other loci in linkage disequilibrium with *ospC* are likely involved in conferring host-adapted phenotypes via multiple interrelated mechanisms (87). Our results suggest genotype–host associations may be partially due to variation at *ospC* directly impacting fitness across host immune environments and should be a target of further investigation.

Studies of *ospC* variation consistently report <2 and >8% nucleotide variation within and among all genotypes, respectively (31, 39, 88). This bimodal distribution is expected to reflect antigenic interactions, whereby different genotypes display distinct epitopes that limit cross-reactivity of host antibodies (56, 89). Interestingly, the genotype identified here as type C_J_ does not conform to this widespread trend, exhibiting a 2.7% nucleotide difference from type C. Our recombination analysis indicated that genotype type C_J_ is the product of the incorporation of the entire type J 4th α-helix (residues 158–183) into type C. A similar variant (type C_KR10_) was previously reported circulating in tick populations in both the Northeastern and Midwestern United States (90). In our study, type C_J_ exhibits a decreased association with white-footed mice compared with type C. Together, these patterns suggest that selection otherwise maintaining coinfection with divergent types is relaxed for this genotype, setting the stage for further investigation of the mechanisms involved and the role of differential host adaptation.

## Methods

### Study site and sample collection

Nymphal ticks were collected on Block Island, RI, USA, between May and August, from 2014 to 2020. Small mammals, mainly white-footed mice, were trapped in the same months from 2014 to 2019, and birds were sampled in the same months from 2013 to 2019. Grids were established for tick and small mammal sampling at three sites across the island at the Block Island National Wildlife Refuge (NR), a private property on the eastern part of the island (EI), and Rodman's Hollow (RH). Passerine birds were mist-netted at two sites at the Ocean View Pavilion and Bayrose Cabin by Kim Gaffett (US Department of Interior Banding Permit 09636). Nymphal ticks were collected from each grid by a standard 1-m^2^ drag cloth method (91), stopping every 10 m to remove attached ticks, which were immediately stored in 70% v/v ethanol until DNA extraction. Small mammals were trapped biweekly at each site for seven sessions consisting of three consecutive trap nights each, using Sherman live traps in accordance with approved Columbia University IACUC protocol AC-AAAS6470 and scientific collection permits from Rhode Island Department of Environmental Management and town of New Shoreham, RI. Block Island supports a low-diversity small mammal community (92) dominated by *Peromyscus leucopus* (white-footed mouse), with <1% other hosts (75). Upon capture morphological characteristics, such as body, tail, and foot measurements, weight, sex, age, and reproduction status were recorded. Each animal was carefully examined for ticks and other ectoparasites and marked with a uniquely numbered ear tag. Ear biopsy tissue and engorged *I. scapularis* larvae removed from all birds and stored in 70% v/v ethanol until DNA extraction and subsequent pathogen analysis.

### DNA extraction, amplification, and sequencing

Genomic DNA was extracted using the DNeasy Blood and Tissue kit optimized for QIAcube HT automation (Qiagen). Questing nymphal and engorged larval ticks from birds were dried, frozen with liquid nitrogen, and crushed with sterile pestles before extraction. Mouse ear tissue was processed using the manufacturer's optimized protocol. Individual samples were then screened using duplex quantitative PCR for *B. burgdorferi* and *Borrelia miyamotoi* in duplicate on an ABI 7500 Fast Real-Time PCR System using TaqMan Fast Advanced Master Mix (Thermo Fisher Scientific).

For *Bb*-positive samples, we used a standard PCR to amplify a region centered around the *ospC* locus. Amplification was first attempted for a 1500-bp region with the following primers 5′-GGGATCCAAAATCTAATACAA-3′ (forward) and 5′-CCCTTAACATACAATATCTCTTC-3′ (reverse). If samples failed to amplify due to DNA degradation, we attempted amplification of a smaller 750 bp region that contained the entire *ospC* locus using the following primers 5′-GAGGCACAAATTAATGAAAAAGAA (forward) and GACTTTATTTTTCCAGTTACTTTTT-3′ (reverse). Both primer sets were designed to target conserved sequences identified across 18 *Bb* genotypes downloaded from GenBank (accession codes provided in Table S8). The PCR protocol included 30 s of denaturation at 98°C followed by 35 cycles of denaturation at 98°C for 10 s, annealing at 60°C for 10 s, and extension at 72°C for 1 min for the longer fragment or 30 s for the shorter fragment, followed by a final extension for 5 min at 72°C.

Each sample was amplified using a unique set of barcoded forward and reverse primers to enable pooling and downstream sample demultiplexing (Tables S9 and S10). Amplification success was assessed by running 2 µl PCR product aliquots on a 1.5% w/v agarose gel. Successful PCR products were cleaned with the ProNex Size-Selective Purification System (Promega) then quantified with a Qubit 3.0 Fluorometer using the dsDNA High Sensitivity Assay kit (Thermo Fisher Scientific) or Fragment Analyzer using the HS NGS Fragment kit (Agilent). Products were pooled at equimolar concentrations, and libraries were constructed for sequencing using the single-molecule real-time (SMRTbell) sequencing Express Template Prep Kit 2.0 with an extended DNA damage repair step, then sequenced with the Pacific Biosciences Sequel I platform at the Icahn School of Medicine at Mt Sinai Genomics Core Facility (New York, NY).

### Sequence clustering and identification of genotypes

High fidelity (HiFi) reads were generated from subreads of each zero-mode waveguide using the circular consensus sequencing (*ccs*) tool (https://github.com/PacificBiosciences/ccs) and demultiplexed using *Lima* v2.2.0 (https://github.com/PacificBiosciences/barcoding) with the following parameters: –ccs –dump-removed –dump-clips –guess 80 –min-score 80 –split –output-handles 600. To identify the distribution of *Bb* genotypes among samples, we first created a FASTA containing all demultiplexed reads and used the *PROKKA* gene annotation software (93) to isolate full-length gene sequences. We used *VSEARCH* v2.18.0 toolkit (94), a free and open-source version of the popular *USEARCH* OTU clustering software, to cluster all *ospC* sequences and 32 reference sequences with known *ospC* types (Table S11), using –id 0.98. Of 21 read clusters with a minimum of 200 reads (all other clusters contained <5 reads), 18 clustered with known *ospC* types. We subjected the centroid sequences representing any genotypes without known matches to BLASTN against the full GenBank nucleotide nr database for further identification. Any novel genotype with >8% nucleotide dissimilarity to known genotypes was named according to the sequential list of all known genotypes (i.e. starting with J3), while those with <8% nucleotide dissimilarity were given a subtype designation according to their closest match (i.e. type C_J_). A novel genotype most closely matching the *ospC* of *B. kurtenbachii* was named simply “*B.kurt.*” but not given a designated type name. Finally, we used *SRST2* (95) to assign reads from each demultiplexed sample represented by a minimum of 20 HiFi reads to one of the 21 identified *ospC* types using the following parameters: –min_depth 1 –prob_err 0.005 –max_divergence 2.5.

### Statistical analyses

Statistical analyses and data filtering were conducted in R v4.1.1 unless otherwise noted (96). For each sampled host or nymphal tick, we first filtered any genotype represented by less than three individual HiFi reads. We then ranked transformed genotype abundances for each sample, as PCR-based amplification preferentially amplifies the most common substrate, skewing relative differences between amplified products. We evaluated diversity profiles across mammal, bird, and nymph genotype communities using Hill numbers and further characterized the extent of genotype overlap between genotype communities with the Sorensen similarity index, both implemented with the *SpadeR* R package (97). We also examined correlations between sequencing depth and genotype richness across samples using linear models.

Because the *ospC* locus is heavily influenced by recombination, individual phylogenetic trees do not accurately represent the evolutionary relationships among genotypes. Thus, we built a phylogenetic network using Neighbor-Net (98) after first aligning representative full length *ospC* sequences from each genotype (i.e. centroids of *USEARCH* clusters) as translated amino acids with *MAFFT* (99).

We investigated patterns of co-occurrence between all pairwise genotype combinations across individual mice, birds, and ticks, separately using the *cooccur* R package, which evaluates the predicted and observed probability of genotype co-occurrence within individuals given their abundances in the data set (100). To better interpret patterns of co-occurrence among genotypes, we built linear models in which either nucleotide dissimilarity, as calculated by *Clustal-Omega* (101), or phylogenetic network distance was used to predict the effect size for each pair of genotypes.

We tested for evidence of recombination among genotypes using the *RDP5* analysis suite across 30-bp sliding windows using the following methods: RDP, GENECONV, BOOTSCAN, MAXCHI, CHIMERA, and SISCAN. Putative breakpoints were accepted if detected independently across three or more methods (102).

To test for evidence of host-adapted associations between specific genotypes and mammalian or avian hosts, we used binomial (logarithmic) GLMs, specifying infection with an individual genotype as the response and sample type (i.e. mammal, bird, or nymph) as the predictor, setting “bird” as the reference to highlight differences between birds and mice. To account for detection bias introduced at low sequencing depth, we included an offset term for samples with <100 reads (otherwise set to 1; Fig. S1). A genotype was considered host adapted if it displayed a significant association (*α* = 0.05) with mammalian or avian hosts. We visualized differences in genotype communities among hosts and ticks using NMDS with the *metaMDS* function from the *vegan* R package (103). To examine differences among bird species, we conducted binomial GLMs as described above, but restricted bird samples to species with sample size >10 (Carolina wren: *n* = 28; common yellowthroat: *n* = 20; American robin: *n* = 16; all others ≤8) and included species as the predictor variable, setting “mouse” as the reference.

To test for evidence of temporal variation in genotype frequency, as expected under NFDS, we first calculated the frequency of each genotype within each sample type for each year, then used boxplots to visualize the distributions. We also plotted changes in frequency over time for each genotype at each site. To assess changes in community structure, we used ANOSIM tests from the *vegan* R package (103). For each sample type, communities across each year were compared using Bray–Curtis dissimilarity matrices and run with 999 permutations. If this global test was significant, we tested pairwise yearly combinations for post-hoc identification of significant pairs. We visualized differences in genotype communities by year using NMDS, as described above.

To evaluate the dynamics of genotype community transitions and persistence at the individual host scale, we examined mice that were sampled multiple times within a single year (*n* = 383), using a MSM model, implemented with the *msm* R package (104). We established three infection states reflecting whether the mouse exhibited no *Bb* infection, *Bb* infection dominated (i.e. read depth rank = 1) by a mouse-associated genotype (types C, E3, H, and K), or *Bb* infection dominated by a nonmouse-associated genotype. We estimated the transition rates between state pairs by maximum likelihood using “nlm” optimization. We then extracted transition probabilities over a 4-week period, the mean sojourn time (i.e. persistence) for each state, and the probability of the next state given each starting state. To understand which factors influence genotype community changes, we also used a binomial generalized linear mixed model to model the probability of a change in identity for a mouse's dominant genotype as a function of time (days since first sampling), genotype richness, sex, maturity, and tick burden, using the site and year of capture as random variables.

## Supplementary Material

pgad234_Supplementary_Data

## Data Availability

Nucleotide sequences were accessioned on NCBI under BioProject record PRJNA854978.
