# Supplementary material for: Host adaptation drives genetic diversity in a vector-borne disease system
Source: PNAS Nexus. 2023 Aug 8;2(8):pgad234. doi: 10.1093/pnasnexus/pgad234 (PMC10408703; doi:10.1093/pnasnexus/pgad234)
Supplement: pgad234_Supplementary_Data [file pgad234_supplementary_data.docx]

**Supplementary Information for**

Host adaptation drives genetic diversity in a vector-borne disease system

Matthew A. Combs^1,6,9^, Danielle M. Tufts^1,2^, Ben Adams^3^, Yi-Pin Lin^4,5^, Sergios-Orestis Kolokotronis^6-9^*^,^ Maria A. Diuk-Wasser^1^*

^1^ Department of Ecology, Evolution and Environmental Biology, Columbia University, New York, NY 10027

^2^ Infectious Diseases and Microbiology Department, University of Pittsburgh, Pittsburgh, PA 15261

^3^ Department of Mathematical Sciences, University of Bath, Bath, BA27AY, United Kingdom

^4^ Division of Infectious Diseases, Wadsworth Center, New York State Department of Health, Albany, NY 12201

^5^ Department of Biomedical Sciences, University at Albany, Albany, NY 12203

^6^ Department of Epidemiology and Biostatistics, School of Public Health, SUNY Downstate Health Sciences University, Brooklyn, NY 11203-2098

^7^ Division of Infectious Diseases, Department of Medicine, College of Medicine, SUNY Downstate Health Sciences University, Brooklyn, NY 11203-2098

^8^ Department of Cell Biology, College of Medicine, SUNY Downstate Health Sciences University, Brooklyn, NY 11203-2098

^9^ Institute for Genomics in Health, SUNY Downstate Health Sciences University, Brooklyn, NY 11203-2098

* Corresponding authors:

Sergios-Orestis Kolokotronis, [sok@downstate.edu](mailto:sok@downstate.edu)

Maria Diuk-Wasser, [mad2256@columbia.edu](mailto:mad2256@columbia.edu)

**This PDF file includes:**

Supplementary text

SI References

Figures S1 to S9

Tables S1 to S11

Supplementary Information Text

**Materials & Methods**

**Study site and sample collection**

Nymphal ticks were collected on Block Island, RI, USA, between May and August, 2014-2020. Small mammals, mainly white-footed mice, were trapped in the same months in the period 2014-2019, and birds were sampled in the same months in the period 2013-2019. Grids were established for tick and small mammal sampling at three sites across the island at the Block Island National Wildlife Refuge (NR), a private property on the eastern part of the island (EI), and at Rodman’s Hollow (RH). Passerine birds were mist netted at two sites at the Ocean View Pavilion (OVP) and Bayrose cabin (BR) under the supervision of Kim Gaffett (U.S. Department of Interior Banding Permit #09636). Nymphal ticks were collected from each grid by a standard 1m^2^ drag cloth method (1) stopping every 10m to remove attached ticks which were immediately stored in 70% ethanol until DNA extraction. Small mammals were trapped biweekly at each site for seven sessions consisting of three consecutive trap nights each, using Sherman live traps in accordance with approved Columbia University IACUC protocol (AC-AAAS6470) and scientific collections permits from Rhode Island Department of Environmental Management and town of New Shoreham, RI. Block Island supports a low diversity small mammal community (2) dominated by *Peromyscus leucopus* (white-footed mouse), with <1% other hosts (3). Upon capture morphological characteristics, such as body, tail, and foot measurements, weight, sex, age, and reproduction status were obtained. Each animal was carefully examined for ticks and other ectoparasites and marked with a uniquely numbered ear tag. Ear biopsy tissue was collected and stored in 70% ethanol until DNA extraction and pathogen analysis. Engorged *I. scapularis* larvae were removed from all bird species and stored in 70% v/v ethanol until DNA extraction and subsequent pathogen analysis.

**PCR amplification and long-read deep sequencing**

Genomic DNA was extracted using the DNeasy Blood & Tissue kit optimized for QIAcube HT automation (Qiagen). Questing nymphal and engorged larval ticks from birds were dried, frozen with liquid nitrogen, and crushed with sterile pestles before extraction. Mouse ear tissue was processed using the manufacturer’s optimized protocol. Individual samples were then screened using a duplex quantitative PCR for *Borrelia burgdorferi* and *Borrelia miyamotoi* in duplicate on an ABI 7500 Fast Real-Time PCR System using TaqMan Fast Advanced Master Mix (Thermo Fisher Scientific).

For Bb-positive samples, we used a standard PCR to amplify a region centered around the *ospC* locus. Amplification was first attempted for a 1500bp region with the following primers GGGATCCAAAATCTAATACAA (forward) and CCCTTAACATACAATATCTCTTC (reverse). If samples failed to amplify due to DNA degradation, we attempted amplification of a smaller 750bp region that contained the entire *ospC* locus using the following primers GAGGCACAAATTAATGAAAAAGAA (forward) and GACTTTATTTTTCCAGTTACTTTTT (reverse). Both primer sets were designed to target conserved sequences identified across 18 *Bb* genotypes downloaded from GenBank (accession codes provided in Table S8). The PCR protocol included 30 s of denaturation at 98°C followed by 35 cycles of denaturation at 98°C for 10 s, annealing at 60°C for 10 s, and extension at 72°C for 1 min for the longer fragment or 30 s for the shorter fragment, followed by a final extension for 5 min at 72°C.

Each sample was amplified using a unique set of barcoded forward and reverse primers to enable pooling and downstream sample demultiplexing (Tables S9, S10). Amplification success was assessed by running 2 µl PCR product aliquots on a 1.5% agarose gel. Successful PCR products were cleaned with the ProNex Size-Selective Purification System (Promega) then quantified with a Qubit 3.0 Fluorometer using the dsDNA High Sensitivity Assay kit (Thermo Fisher Scientific) or Fragment Analyzer using the HS NGS Fragment kit (Agilent). Products were pooled at equimolar concentrations and libraries were constructed for sequencing using the single-molecule real-time (SMRTbell) sequencing Express Template Prep Kit 2.0 with an extended DNA damage repair step, then sequenced with the Pacific Biosciences Sequel I platform at the Genomics Core Facility of the Icahn School of Medicine at Mt. Sinai (New York, NY).

**Sequence clustering and identification of genotypes**

High fidelity (HiFi) reads were generated from subreads of each zero-mode waveguide (ZMW) using the circular consensus sequencing (*ccs*) tool (<https://github.com/PacificBiosciences/ccs>) and demultiplexed using *Lima* v2.2.0 (<https://github.com/PacificBiosciences/barcoding>) with the following parameters: --ccs --dump-removed --dump-clips --guess 80 --min-score 80 --split --output-handles 600. To identify the distribution of *Bb* genotypes among samples we first created a FASTA containing all demultiplexed reads and used *PROKKA* for gene annotation (4) to isolate full-length gene sequences. We used *VSEARCH* v2.18.0 toolkit (5), a free and open-source version of the popular *USEARCH* OTU clustering software, to cluster all sequenced *ospC* loci and 32 reference sequences with known *ospC* types (Table S11), using --id 0.98. Of 21 read clusters with a minimum of 200 reads (all other clusters contained <5 reads), 18 clustered with known *ospC* types. We subjected the centroid sequences representing any genotypes without known matches to a *BLASTN* search against the full Genbank nucleotide *nr* database for further identification. Any novel genotype with >8% nucleotide dissimilarity to known genotypes was named according to the sequential list of all known genotypes (i.e. starting with J3), while those with <8% nucleotide dissimilarity were given a subtype designation according to the closest match (i.e. type C_J_). A novel genotype most closely matching the *ospC* of *Borrelia kurtenbachii* was named simply “B.kurt.”, but was not given a designated type name. Finally, we used *SRST2* (6) to assign reads from each demultiplexed sample represented by a minimum of 20 HiFi reads to one of the 21 identified *ospC* types using the following parameters: --min_depth 1 --prob_err 0.005 --max_divergence 2.5.

**Statistical analyses**

*ospC* nucleotide sequences were accessioned on NCBI under BioProject record PRJNA854978.

Statistical analyses and data filtering were conducted in R v4.1.1 unless otherwise noted (R Core Team 2021). For each sampled host or nymphal tick, we first filtered any genotype represented by less than three individual HiFi reads. We then rank transformed genotype abundances for each sample, as PCR-based amplification preferentially amplifies the most common substrate, skewing relative differences between amplified products. We evaluated diversity profiles across mammal, bird, and nymph genotype communities using Hill numbers and further characterized the extent of genotype overlap between genotype communities with the Sørensen index, both implemented in the *SpadeR* R package (8). We also examined correlations between sequencing depth on genotype richness across samples using linear models.

Because the *ospC* locus is heavily influenced by recombination, individual phylogenetic trees do not accurately represent the evolutionary relationships among genotypes. Thus, we built a phylogenetic network using Neighbor-Net (9) after first aligning representative full length *ospC* sequences from each genotype (i.e. centroids of USEARCH clusters) as translated amino acids with *MAFFT* (10).

We investigated patterns of co-occurrence between all pairwise genotype combinations across individual mice, birds, and ticks, separately using the *cooccur* R package, which evaluates the predicted and observed probability of genotype co-occurrence within individuals given their abundances in the dataset (11). To better interpret patterns of co-occurrence among genotypes we built linear models in which either nucleotide dissimilarity, as calculated by Clustal-Omega (12), or phylogenetic network distance, was used to predict the effect size for each pair of genotypes.

We tested for evidence of recombination among genotypes using the *RDP5* analysis suite across 30bp sliding windows using the following methods: *RDP*, *GENECONV*, *BOOTSCAN*, *MAXCHI*, *CHIMERA*, *SISCAN*. Putative breakpoints were accepted if detected with three or more methods independently (13).

To test for evidence of host-adapted associations between specific genotypes and mammalian or avian hosts we used binomial (logarithmic) generalized linear models (GLMs), specifying infection with an individual genotype as the response and sample type (i.e. mammal, bird, or nymph) as the predictor, setting “bird” as the reference to highlight differences between birds and mice. To account for detection bias introduced at low sequencing depth we included an offset term for samples with <100 reads (otherwise set to 1; Figure S1). A genotype was considered host-adapted if it displayed a significant association (α = 0.05) with mammalian or avian hosts. We visualized differences in genotype communities among hosts and ticks using nonmetric multidimensional scaling (NMDS) with the *metaMDS* function from the *vegan* R package (14). To examine differences among bird species, we conducted binomial GLMs as described above, but restricted bird samples to species with sample size greater than 10 (Carolina wren: *n* = 28, Common yellowthroat: *n* = 20, American robin: *n* = 16, all others ≤ 8) and included species as the predictor variable, setting “mouse” as the reference.

To test for evidence of temporal variation in genotype frequency, as expected under NFDS, we first calculated the frequency of each genotype within each sample type for each year, then used boxplots to visualize the distributions. We also plotted changes in frequency over time for each genotype at each site. To assess changes in community structure we used Analysis of Similarities (ANOSIM) tests from the *vegan* R package (14). For each sample type, communities across each year were compared using Bray-Curtis dissimilarity matrices and run with 999 permutations. If this global test was significant, we tested pairwise yearly combinations for post-hoc identification of significant pairs. We visualized differences in genotype communities by year using NMDS as described above.

To evaluate the dynamics of genotype community transitions and persistence at the individual host scale we examined mice that were sampled multiple times within a single year (n = 383) using a multi-state Markov (MSM) model, implemented with the *msm* R package (15). We established three infection states reflecting whether the mouse exhibited no *Bb* infection, *Bb* infection dominated (i.e. read depth rank = 1) by a mouse-associated genotype (type C, E3, H, K), or *Bb* infection dominated by a non-mouse-associated genotype. We estimated the transition rates between state pairs by maximum likelihood using ‘nlm’ optimization. We then extracted transition probabilities over a 4-week period, the mean sojourn time (i.e. persistence) for each state, and the probability of the next state given each starting state. To understand what factors influence genotype community changes we also used a binomial generalized linear mixed model (GLMM) to model the probability of a change in identity for a mouse’s dominant genotype as a function of time (days since first sampling), genotype richness, sex, maturity, and tick burden, using the site and year of capture as random variables.

**SI References**

1. R. C. Falco, D. Fish, A comparison of methods for sampling the deer tick, *Ixodes dammini*, in a Lyme disease endemic area. *Exp. Appl. Acarol.* **14**, 165–173 (1992).

2. S. B. Comings, *The Nature of Block Island* (Royal Bruce Inc., 2006).

3. C. I. Huang, *et al.*, High burdens of *Ixodes scapularis* larval ticks on white-tailed deer may limit Lyme disease risk in a low biodiversity setting. *Ticks Tick. Borne. Dis.* **10**, 258–268 (2019).

4. T. Seemann, Prokka: Rapid prokaryotic genome annotation. *Bioinformatics* **30**, 2068–2069 (2014).

5. T. Rognes, T. Flouri, B. Nichols, C. Quince, F. Mahé, VSEARCH: A versatile open source tool for metagenomics. *PeerJ* **2016**, 1–22 (2016).

6. M. Inouye, *et al.*, SRST2: Rapid genomic surveillance for public health and hospital microbiology labs. *Genome Med.* **6**, 1–16 (2014).

7. R. C. Team, R: A Language and environment for statistical computing (2021).

8. A. Chao, K. H. Ma, T. C. Hsieh, C.-H. Chiu, SpadeR: species-richness prediction and diversity estimation with R. *CRAN* (2016).

9. D. Bryant, V. Moulton, Neighbor-Net: An agglomerative method for the construction of phylogenetic networks. *Mol. Biol. Evol.* **21**, 255–265 (2004).

10. K. Katoh, D. M. Standley, MAFFT multiple sequence alignment software version 7: Improvements in performance and usability. *Mol. Biol. Evol.* **30**, 772–780 (2013).

11. D. M. Griffith, J. A. Veech, C. J. Marsh, Cooccur: Probabilistic species co-occurrence analysis in R. *J. Stat. Softw.* **69**, 1–17 (2016).

12. F. Sievers, D. G. Higgins, Clustal Omega. *Curr. Protoc. Bioinforma.* **2014**, 3.13.1-3.13.16 (2014).

13. D. P. Martin, *et al.*, RDP5: A computer program for analyzing recombination in, and removing signals of recombination from, nucleotide sequence datasets. *Virus Evol.* **7**, 5–7 (2021).

14. A. J. Oksanen, *et al.*, Vegan: Community Ecology Package. *CRAN* (2020) https:/doi.org/10.1007/978-94-024-1179-9_301576.

15. C. H. Jackson, Multi-state models for panel data: The msm package for R. *J. Stat. Softw.* **38**, 1–28 (2011).


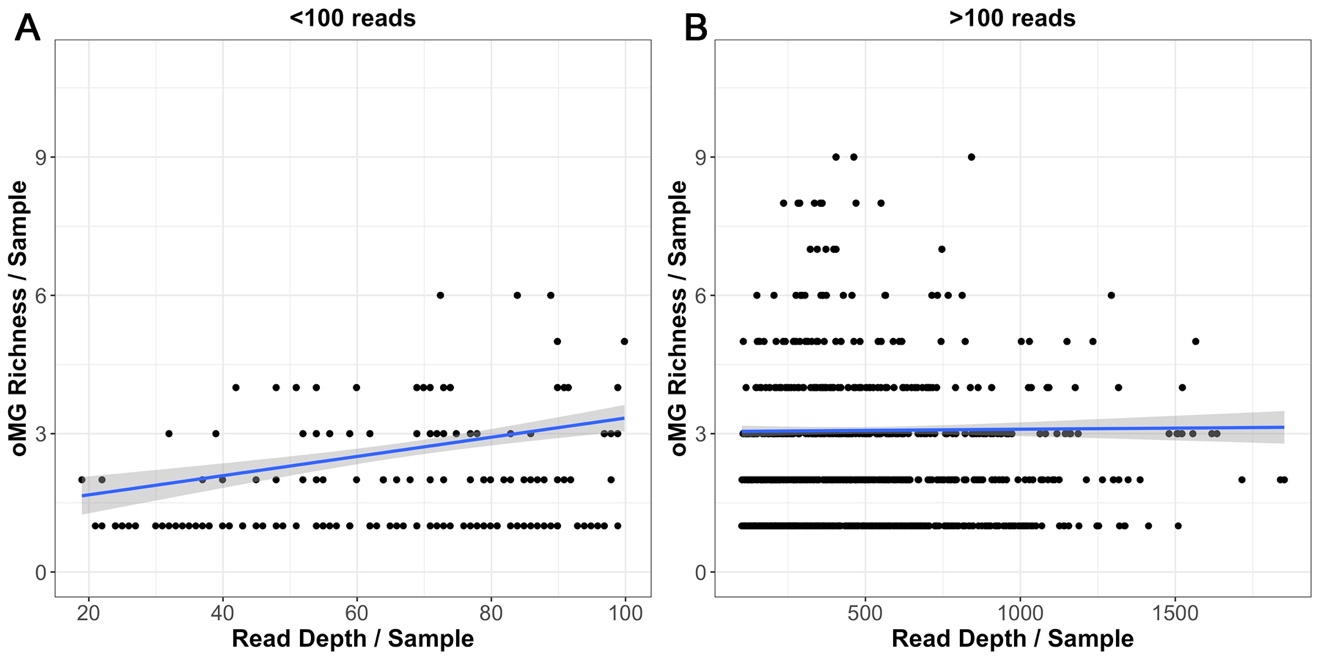


Fig. S1. Sequencing depth bias observed for samples represented by fewer than 100 reads (A) but absent for samples represented by more than 100 reads (B).


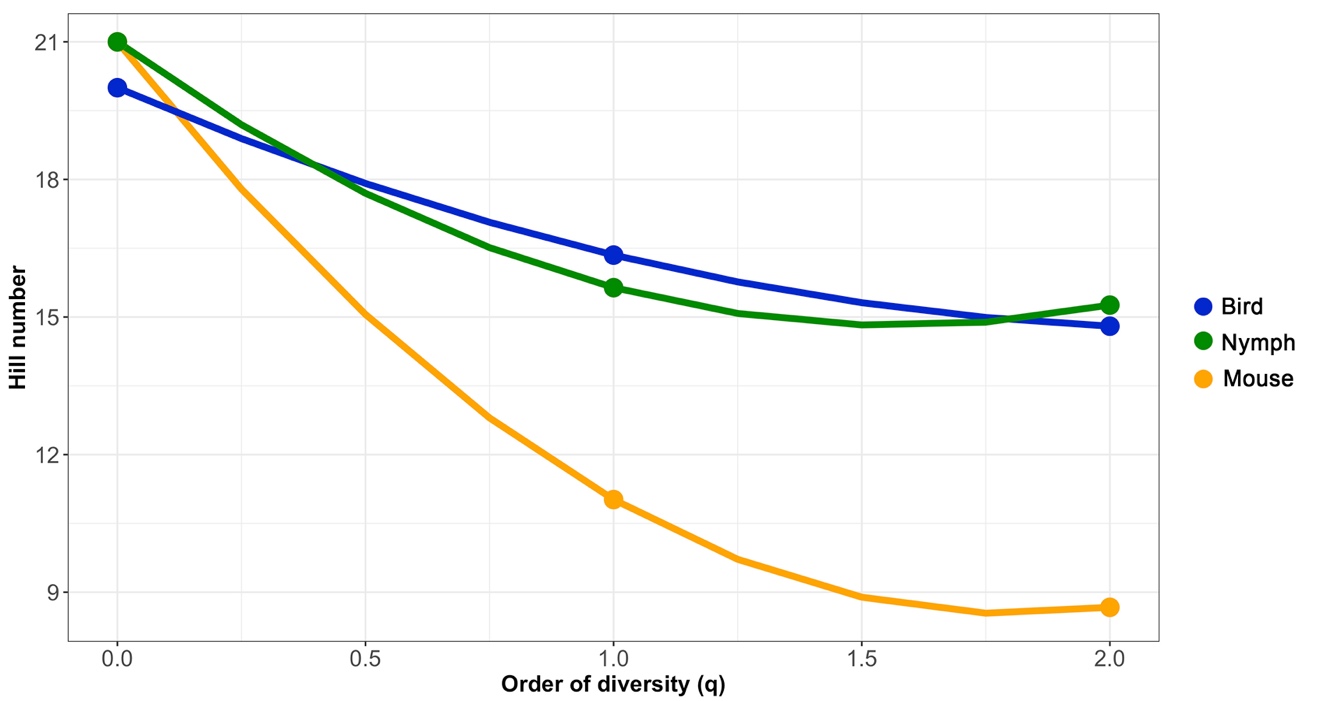


Fig. S2. Diversity profiles using Hill numbers for genotype communities observed within mice (orange), birds (blue), or nymphs (green). Flatter profiles represent greater evenness and steeper slopes represent increasing unevenness. q = 0 is equivalent to community richness, while q = 1 is equivalent to the Shannon index of diversity, and q = 2 is equivalent to the multiplicative inverse of the Simpson diversity index.


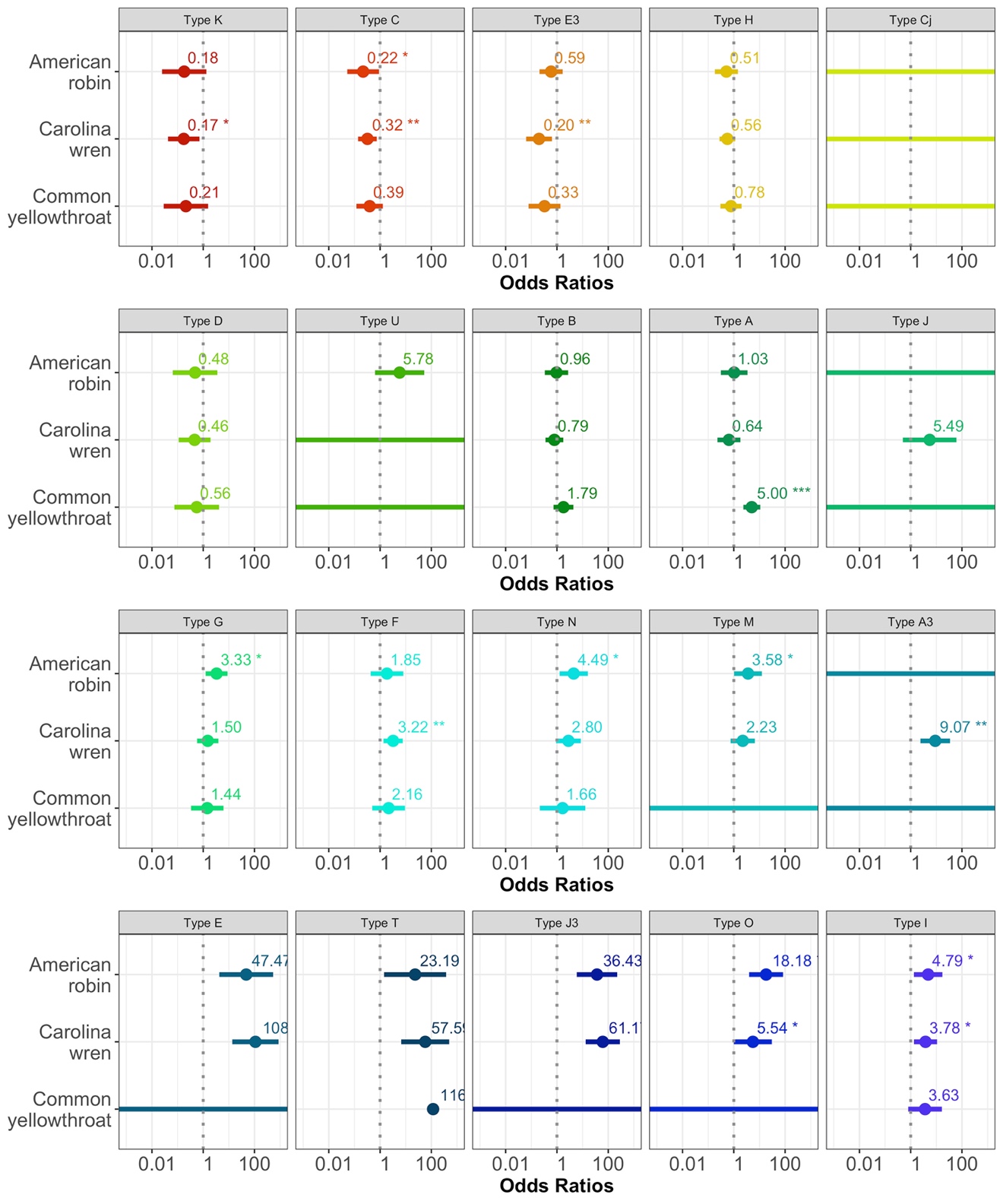


**Fig. S3.** Bird species-specific binomial GLMs for each *Bb* genotype. Odds ratios are relative to genotype infection in mice. Colors represent strength of host association observed when bird species were treated as a single taxa group, as labeled in Fig. 1.

**
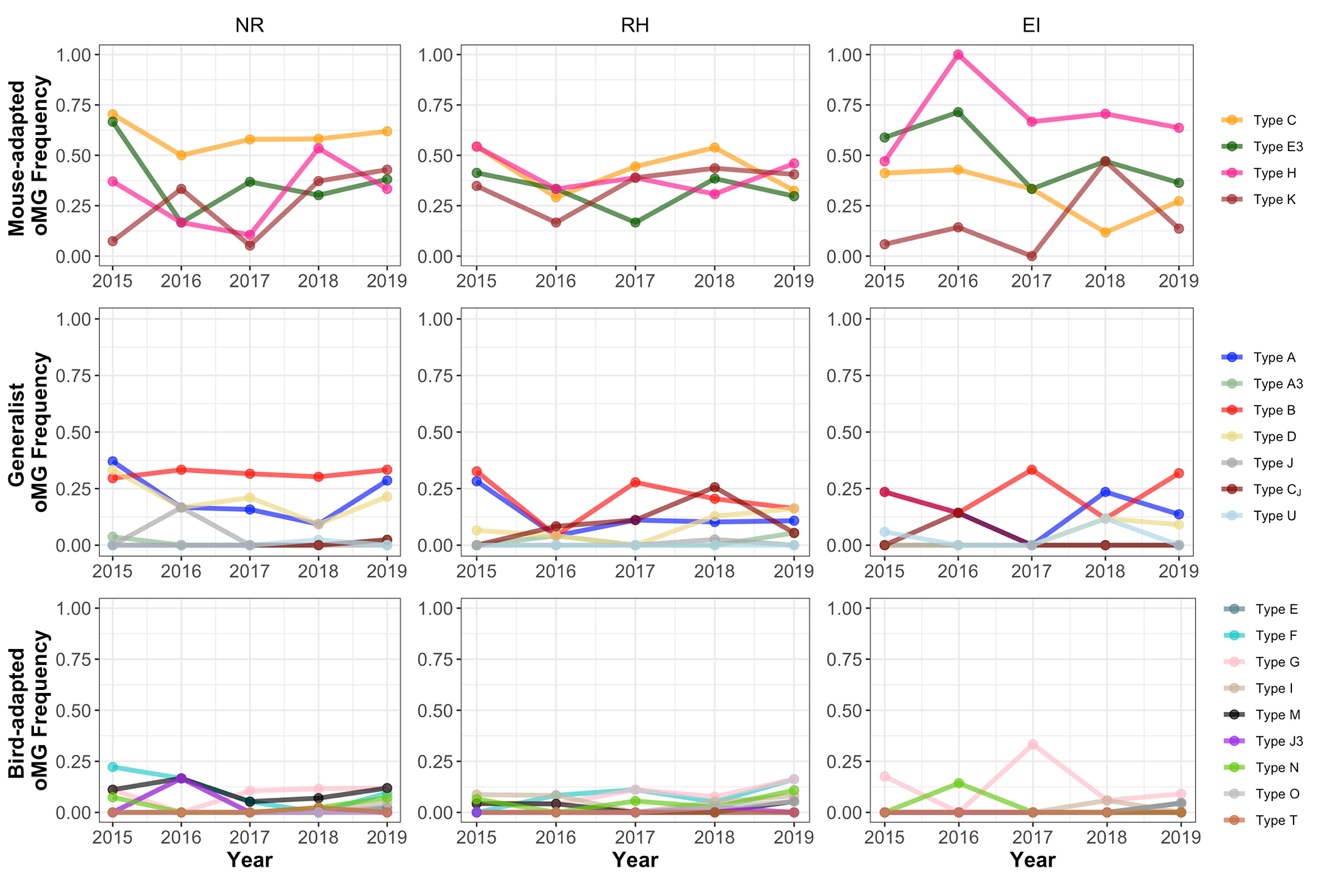
**

**Fig. S4.** Yearly frequency of individual genotypes in mice across sites.

**
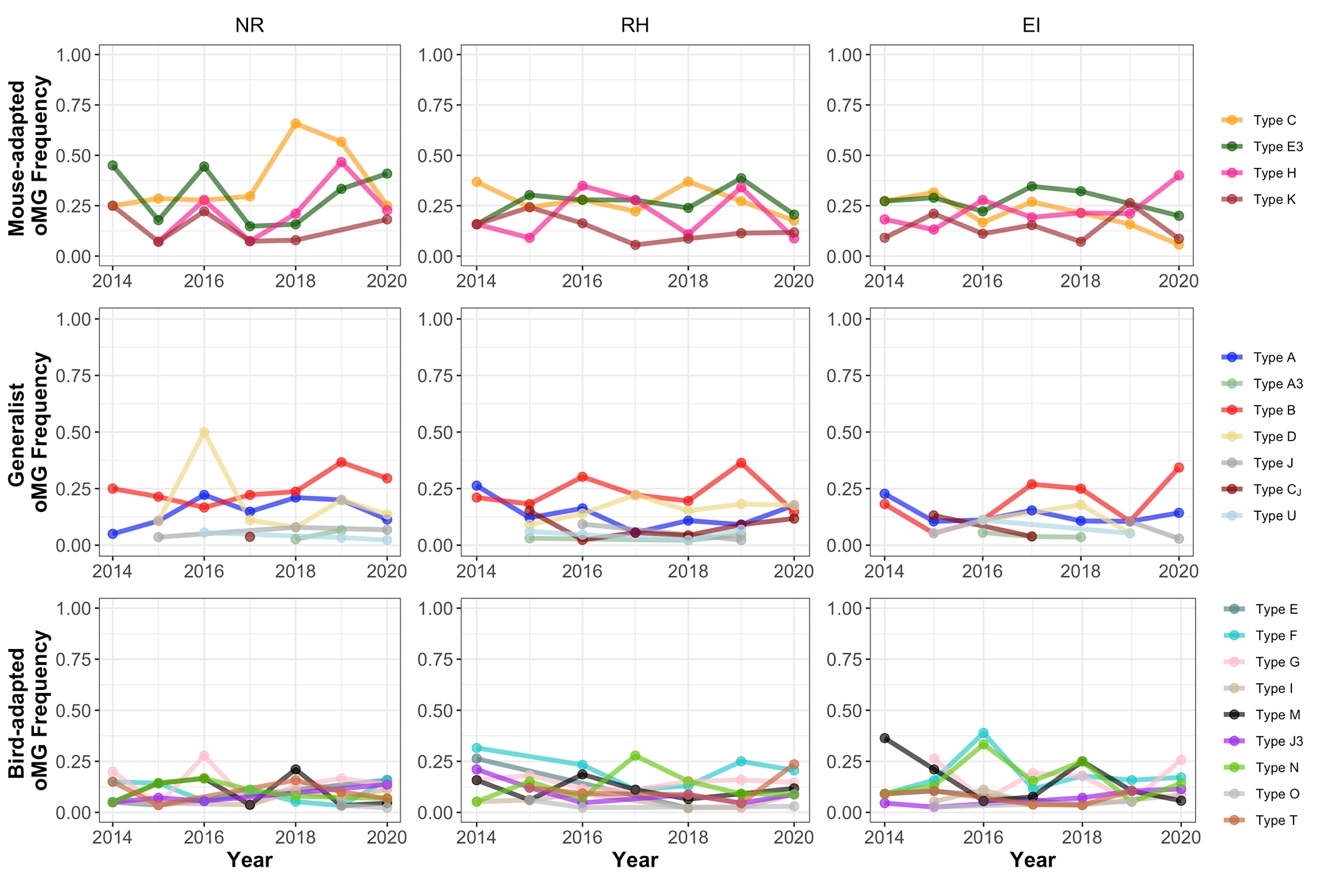
**

**Fig. S5.** Yearly frequency of individual genotypes in nymphal ticks across sites.

**
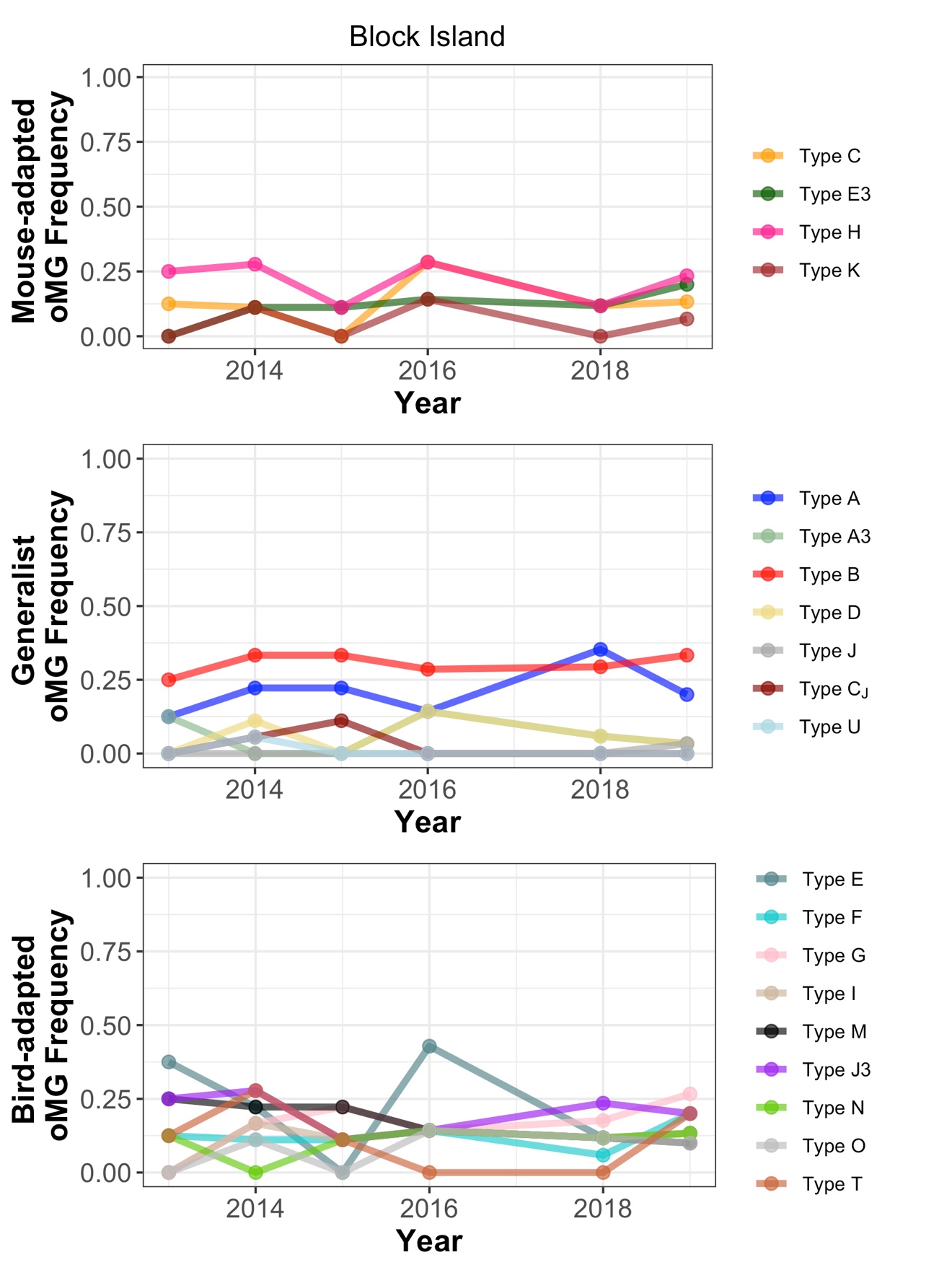
**

**Fig. S6.** Yearly frequency of individual genotypes in bird hosts, excluding 2017 where n = 2.


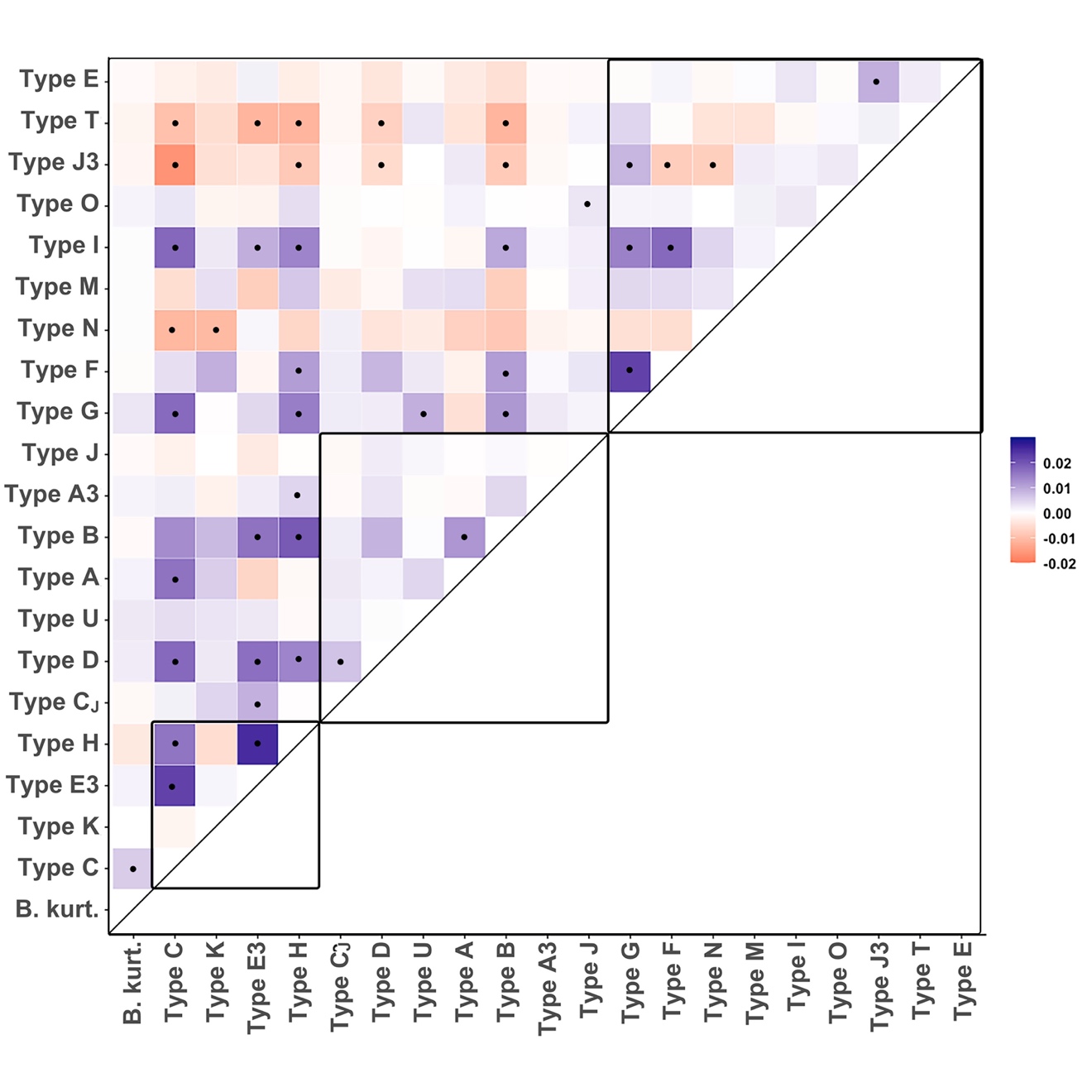


**Fig. S7.** Heatmap of genotype co-occurrence probability effect sizes for nymphal ticks. Color indicates the strength of the effect (blue = positive, red = negative), and black dots indicate significant associations (*p* < 0.05). Black boxes within the heatmap surround the putative mouse-adapted genotypes (bottom), generalist genotypes (middle), and bird-adapted genotypes (top).


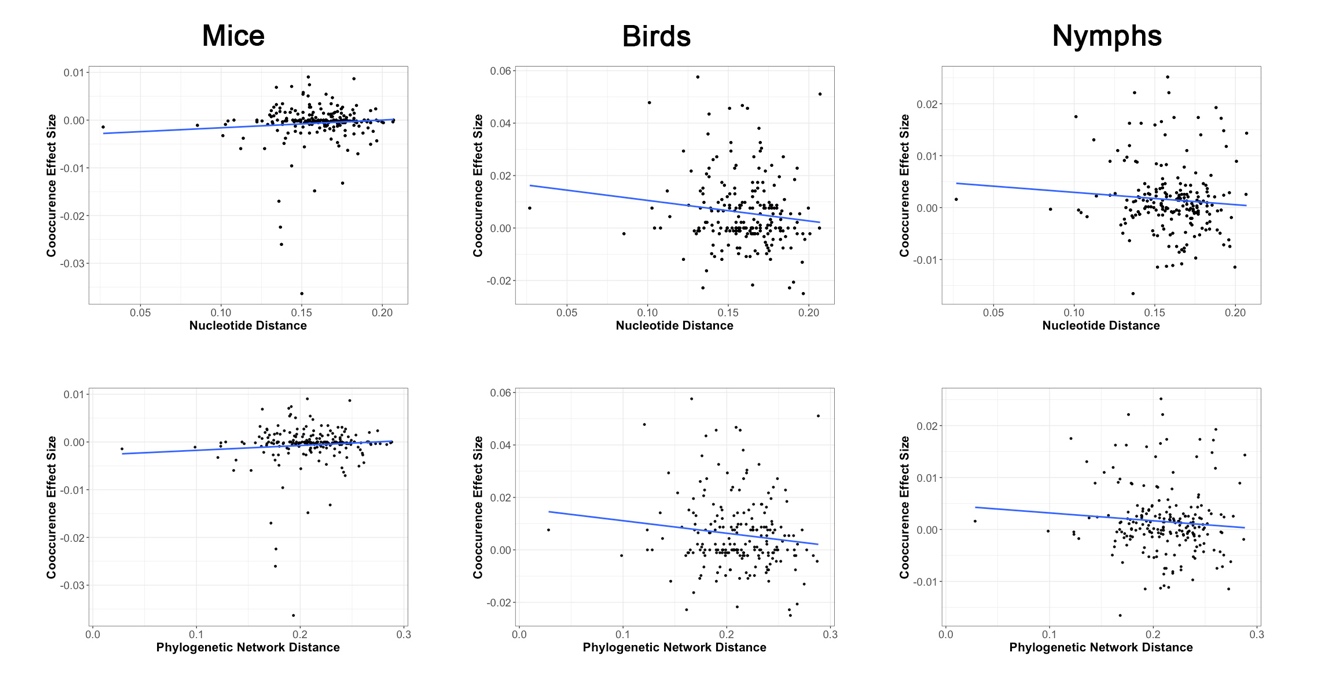


**Fig. S8.** Correlations between pairwise co-occurrence effect sizes among genotypes and nucleotide distance (top row) or phylogenetic network distance (bottom row) among mice (left column), birds (middle column), and nymphal ticks (right column).


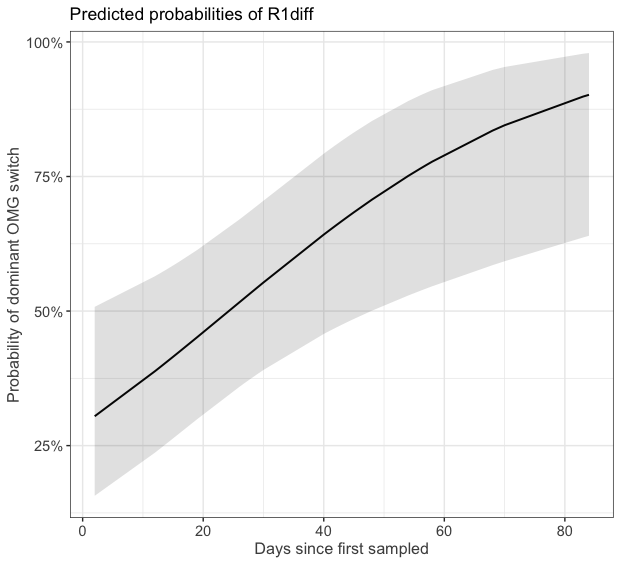


**Fig. S9.** Predicted relationship between time (days) and the probability of turnover in the dominant genotype within individual mammal communities.

Table S1. Sequencing of genotypes across Bb infected tick nymphs, white footed mice, and passerine birds. Details for individual bird species are provided in Table S3.

Table S2. Pairwise genotype community similarity among populations of mammals, birds, and nymphs calculated with the local (Sørenson-type) species overlap measure.

|  | Mammals | Birds | Nymphs |
| --- | --- | --- | --- |
| Mammals | 1.000 | 0.672 | 0.904 |
| Birds |  | 1.000 | 0.883 |
| Nymphs |  |  | 1.000 |

**Table S3.** *Bb* genotypes across individual bird species.

**Table S4.** Mean sojourn time (days) for each infection state as determined by the MSM model. Values represent the expected duration of a mouse infection in days. 95% confidence intervals are provided in parentheses.

|  | Mean sojourn time (days) |
| --- | --- |
| Uninfected | 53.9 (41.3-71.2) |
| Adapted | 27.6 (19.6-38.7) |
| Non-Adapted | 9.5 (6.2-14.5) |

**Table S5.** Estimated probabilities of a mouses next different infection state (rows), given the current state (columns), determined by the MSM model. 95% confidence intervals are provided in parentheses. NA, not available

|  | Uninfected | Adapted | Non-Adapted |
| --- | --- | --- | --- |
| Uninfected | NA | 0.37 (0.16-0.65) | 0.51 (0.35-0.70) |
| Adapted | 0.32 (0.14-0.55) | NA | 0.49 (0.30-0.65) |
| Non-Adapted | 0.68 (0.45-0.86) | 0.63 (0.35-0.84) | NA |

**Table S6.** Model results for the GLMM testing influence of time and individual mouse characteristics on the probability of observing a switch in the dominant genotype.

| Predictors | Odds Ratios | 95% CI | *p*-value |
| --- | --- | --- | --- |
| (Intercept) | 0.51 | 0.20-1.30 | 0.164 |
| Time since first sample (days) | 1.03 | 1.01-1.06 | **0.006** |
| *ospC* type richness | 1.13 | 0.84-1.53 | 0.43 |
| Sex (Male) | 0.92 | 0.50-1.71 | 0.796 |
| Age (non-adult) | 1.52 | 0.28-11.41 | 0.643 |
| Nymphal burden | 0.99 | 0.94-1.06 | 0.862 |

**Table S7.** Recombination analysis results.

| **In Alignment** | | **In Recombinant Sequence** | |  |  |  | **Detection method** | | | | | | |
| --- | --- | --- | --- | --- | --- | --- | --- | --- | --- | --- | --- | --- | --- |
| **Begin** | **End** | **Begin** | **End** | **Recombinant sequence(s)** | **Minor parental sequence(s)** | **Major parental sequence(s)** | **RDP** | **GENECONV** | **Bootscan** | **Maxchi** | **Chimaera** | **SiSscan** | **3Seq** |
| 303 | 413 | 297 | 398 | B | Cj, C | J, H | 5.15E-05 | 2.21E-04 | 2.18E-06 | 1.62E-07 | 1.37E-07 | 1.62E-09 | 1.12E-07 |
| 459 | 554 | 441 | 530 | B.kurt | J | C | NS | 2.84E-07 | 1.28E-08 | 2.56E-04 | 2.56E-04 | 1.63E-05 | 8.66E-08 |
| 350 | 408 | 335 | 393 | I | D | Cj, C | 3.84E-07 | 2.78E-06 | 1.53E-06 | 4.60E-03 | 7.96E-04 | 2.64E-05 | 2.38E-05 |
| 303 | 493 | 297 | 469 | J3 | A | M | NS | 2.38E-03 | 9.61E-04 | 5.91E-06 | 9.63E-03 | 3.89E-07 | 6.14E-07 |
| 142 | 418 | 139 | 403 | A | G | C, Cj | NS | NS | NS | 5.17E-05 | 4.68E-06 | 4.50E-03 | 4.55E-04 |
| 320 | 526 | 317 | 508 | B.kurt | E | H | NS | NS | NS | 1.91E-05 | 4.38E-02 | NS | 1.03E-02 |
| 628 | 186 | 604 | 183 | H | O | B.kurt | NS | 2.22E-03 | 1.96E-03 | 1.88E-02 | 1.43E-02 | NS | 2.22E-03 |
| 518 | 198 | 497 | 195 | E3 | U | G | NS | NS | 8.77E-03 | 2.09E-04 | 5.25E-05 | 4.67E-04 | NS |
| 608 | 294 | 587 | 291 | K | J | N | NS | NS | 8.03E-04 | NS | 5.73E-03 | NS | NS |
| 250 | 309 | 247 | 303 | J3 | E | A | NS | NS | NS | 2.51E-03 | NS | 1.49E-03 | 1.66E-03 |
| 340 | 418 | 326 | 400 | O | C, Cj | J, H | NS | NS | 2.19E-03 | 4.52E-03 | 1.66E-03 | 2.85E-05 | 4.28E-02 |

**Table S8.** GenBank accessions for strains used for nucleotide alignment during primer design.

| Accession | Strain |
| --- | --- |
| CP001550 | 29805 |
| CP002239 | N40 |
| CP001446 | WI91-23 |
| CP017202 | B331 |
| CP001535 | 118a |
| CP001375 | 72a |
| CP001484 | CA-11.2A |
| CP001493 | 94a |
| CP031398 | MM1 |
| CP002316 | JD1 |
| CP001271 | 156a |
| CP002268 | 297 |
| CP001422 | 64b |
| CP001212 | ZS7 |
| AE000792 | B31 |
| CP019917 | Pabe |
| CP019755 | B31_NRZ |
| CP001568 | Bol26 |

**Table S9.** Barcoded *ospC* primer set 1 used in this study to amplify a 1500-bp region centered around the *ospC* locus. Barcodes selected from a set of 384 sequences provided by Pacific Biosciences.

| Primer name | Barcoded primer sequence |
| --- | --- |
| ospC_16.6_F_1 | CACATATCAGAGTGCGGGGATCCAAAATCTAATACAA |
| ospC_16.6_F_2 | ACACACAGACTGTGAGGGGATCCAAAATCTAATACAA |
| ospC_16.6_F_3 | ACACATCTCGTGAGAGGGGATCCAAAATCTAATACAA |
| ospC_16.6_F_4 | CACGCACACACGCGCGGGGATCCAAAATCTAATACAA |
| ospC_16.6_F_5 | CACTCGACTCTCGCGTGGGATCCAAAATCTAATACAA |
| ospC_16.6_F_6 | CATATATATCAGCTGTGGGATCCAAAATCTAATACAA |
| ospC_16.6_F_7 | TCTGTATCTCTATGTGGGGATCCAAAATCTAATACAA |
| ospC_16.6_F_8 | ACAGTCGAGCGCTGCGGGGATCCAAAATCTAATACAA |
| ospC_16.6_F_9 | ACACACGCGAGACAGAGGGATCCAAAATCTAATACAA |
| ospC_16.6_F_10 | ACGCGCTATCTCAGAGGGGATCCAAAATCTAATACAA |
| ospC_16.6_F_11 | CTATACGTATATCTATGGGATCCAAAATCTAATACAA |
| ospC_16.6_F_12 | ACACTAGATCGCGTGTGGGATCCAAAATCTAATACAA |
| ospC_16.6_F_13 | CTCTCGCATACGCGAGGGGATCCAAAATCTAATACAA |
| ospC_16.6_F_14 | CTCACTACGCGCGCGTGGGATCCAAAATCTAATACAA |
| ospC_16.6_F_15 | CGCATGACACGTGTGTGGGATCCAAAATCTAATACAA |
| ospC_16.6_F_16 | CATAGAGAGATAGTATGGGATCCAAAATCTAATACAA |
| ospC_16.6_F_17 | CACACGCGCGCTATATGGGATCCAAAATCTAATACAA |
| ospC_16.6_F_18 | TCACGTGCTCACTGTGGGGATCCAAAATCTAATACAA |
| ospC_16.6_F_19 | ACACACTCTATCAGATGGGATCCAAAATCTAATACAA |
| ospC_16.6_F_20 | CACGACACGACGATGTGGGATCCAAAATCTAATACAA |
| ospC_16.6_F_21 | CTATACATAGTGATGTGGGATCCAAAATCTAATACAA |
| ospC_16.6_F_22 | CACTCACGTGTGATATGGGATCCAAAATCTAATACAA |
| ospC_16.6_F_23 | CAGAGAGATATCTCTGGGGATCCAAAATCTAATACAA |
| ospC_16.6_F_24 | CATGTAGAGCAGAGAGGGGATCCAAAATCTAATACAA |
| ospC_16.6_F_25 | CGCGACACGCTCGCGCGGGATCCAAAATCTAATACAA |
| ospC_16.6_F_26 | CACAGAGACACGCACAGGGATCCAAAATCTAATACAA |
| ospC_16.6_F_27 | CTCACACTCTCTCACAGGGATCCAAAATCTAATACAA |
| ospC_16.6_F_28 | CTCTGCTCTGACTCTCGGGATCCAAAATCTAATACAA |
| ospC_16.6_F_29 | TATATATGTCTATAGAGGGATCCAAAATCTAATACAA |
| ospC_16.6_F_30 | TCTCTCTATCGCGCTCGGGATCCAAAATCTAATACAA |
| ospC_16.6_F_31 | GATGTCTGAGTGTGTGGGGATCCAAAATCTAATACAA |
| ospC_16.6_F_32 | GAGACTAGAGATAGTGGGGATCCAAAATCTAATACAA |
| ospC_16.6_F_33 | TCTCGTCGCAGTCTCTGGGATCCAAAATCTAATACAA |
| ospC_16.6_F_34 | ATGTGTATATAGATATGGGATCCAAAATCTAATACAA |
| ospC_16.6_F_35 | GCGCGCGCACTCTCTGGGGATCCAAAATCTAATACAA |
| ospC_16.6_F_36 | GAGACACGTCGCACACGGGATCCAAAATCTAATACAA |
| ospC_16.6_F_37 | ACACATATCGCACTACGGGATCCAAAATCTAATACAA |
| ospC_16.6_F_38 | GTGTGTCTCGATGCGCGGGATCCAAAATCTAATACAA |
| ospC_16.6_F_39 | CGCACACATAGATACAGGGATCCAAAATCTAATACAA |
| ospC_16.6_F_40 | TGTCATATGAGAGTGTGGGATCCAAAATCTAATACAA |
| ospC-18.1_R_1 | CGCACTCTGATATGTGCCCTTAACATACAATATCTCTTC |
| ospC-18.1_R_2 | CTCACAGTCTGTGTGTCCCTTAACATACAATATCTCTTC |
| ospC-18.1_R_3 | CTCTCACGAGATGTGTCCCTTAACATACAATATCTCTTC |
| ospC-18.1_R_4 | CGCGCGTGTGTGCGTGCCCTTAACATACAATATCTCTTC |
| ospC-18.1_R_5 | ACGCGAGAGTCGAGTGCCCTTAACATACAATATCTCTTC |
| ospC-18.1_R_6 | ACAGCTGATATATATGCCCTTAACATACAATATCTCTTC |
| ospC-18.1_R_7 | CACATAGAGATACAGACCCTTAACATACAATATCTCTTC |
| ospC-18.1_R_8 | CGCAGCGCTCGACTGTCCCTTAACATACAATATCTCTTC |
| ospC-18.1_R_9 | TCTGTCTCGCGTGTGTCCCTTAACATACAATATCTCTTC |
| ospC-18.1_R_10 | CTCTGAGATAGCGCGTCCCTTAACATACAATATCTCTTC |
| ospC-18.1_R_11 | ATAGATATACGTATAGCCCTTAACATACAATATCTCTTC |
| ospC-18.1_R_12 | ACACGCGATCTAGTGTCCCTTAACATACAATATCTCTTC |
| ospC-18.1_R_13 | CTCGCGTATGCGAGAGCCCTTAACATACAATATCTCTTC |
| ospC-18.1_R_14 | ACGCGCGCGTAGTGAGCCCTTAACATACAATATCTCTTC |
| ospC-18.1_R_15 | ACACACGTGTCATGCGCCCTTAACATACAATATCTCTTC |
| ospC-18.1_R_16 | ATACTATCTCTCTATGCCCTTAACATACAATATCTCTTC |
| ospC-18.1_R_17 | ATATAGCGCGCGTGTGCCCTTAACATACAATATCTCTTC |
| ospC-18.1_R_18 | CACAGTGAGCACGTGACCCTTAACATACAATATCTCTTC |
| ospC-18.1_R_19 | ATCTGATAGAGTGTGTCCCTTAACATACAATATCTCTTC |
| ospC-18.1_R_20 | ACATCGTCGTGTCGTGCCCTTAACATACAATATCTCTTC |
| ospC-18.1_R_21 | ACATCACTATGTATAGCCCTTAACATACAATATCTCTTC |
| ospC-18.1_R_22 | ATATCACACGTGAGTGCCCTTAACATACAATATCTCTTC |
| ospC-18.1_R_23 | CAGAGATATCTCTCTGCCCTTAACATACAATATCTCTTC |
| ospC-18.1_R_24 | CTCTCTGCTCTACATGCCCTTAACATACAATATCTCTTC |
| ospC-18.1_R_25 | GCGCGAGCGTGTCGCGCCCTTAACATACAATATCTCTTC |
| ospC-18.1_R_26 | TGTGCGTGTCTCTGTGCCCTTAACATACAATATCTCTTC |
| ospC-18.1_R_27 | TGTGAGAGAGTGTGAGCCCTTAACATACAATATCTCTTC |
| ospC-18.1_R_28 | GAGAGTCAGAGCAGAGCCCTTAACATACAATATCTCTTC |
| ospC-18.1_R_29 | TCTATAGACATATATACCCTTAACATACAATATCTCTTC |
| ospC-18.1_R_30 | GAGCGCGATAGAGAGACCCTTAACATACAATATCTCTTC |
| ospC-18.1_R_31 | CACACACTCAGACATCCCCTTAACATACAATATCTCTTC |
| ospC-18.1_R_32 | CACTATCTCTAGTCTCCCCTTAACATACAATATCTCTTC |
| ospC-18.1_R_33 | AGAGACTGCGACGAGACCCTTAACATACAATATCTCTTC |
| ospC-18.1_R_34 | ATATCTATATACACATCCCTTAACATACAATATCTCTTC |
| ospC-18.1_R_35 | CAGAGAGTGCGCGCGCCCCTTAACATACAATATCTCTTC |
| ospC-18.1_R_36 | GTGTGCGACGTGTCTCCCCTTAACATACAATATCTCTTC |
| ospC-18.1_R_37 | GTAGTGCGATATGTGTCCCTTAACATACAATATCTCTTC |
| ospC-18.1_R_38 | GCGCATCGAGACACACCCCTTAACATACAATATCTCTTC |
| ospC-18.1_R_39 | TGTATCTATGTGTGCGCCCTTAACATACAATATCTCTTC |
| ospC-18.1_R_40 | ACACTCTCATATGACACCCTTAACATACAATATCTCTTC |

**Table S10** Barcoded *ospC* primer set 2 used in this study to amplify a 750-bp region encompassing the *ospC* locus. Barcodes selected from a set of 384 sequences provided by Pacific Biosciences.

| Primer name | Barcoded primer sequence |
| --- | --- |
| BL-1F-BC1 | CACATATCAGAGTGCGGAGGCACAAATTAATGAAAAAGAA |
| BL-1F-BC2 | ACACACAGACTGTGAGGAGGCACAAATTAATGAAAAAGAA |
| BL-1F-BC3 | ACACATCTCGTGAGAGGAGGCACAAATTAATGAAAAAGAA |
| BL-1F-BC4 | CACGCACACACGCGCGGAGGCACAAATTAATGAAAAAGAA |
| BL-1F-BC5 | CACTCGACTCTCGCGTGAGGCACAAATTAATGAAAAAGAA |
| BL-1F-BC6 | CATATATATCAGCTGTGAGGCACAAATTAATGAAAAAGAA |
| BL-1F-BC7 | TCTGTATCTCTATGTGGAGGCACAAATTAATGAAAAAGAA |
| BL-1F-BC8 | ACAGTCGAGCGCTGCGGAGGCACAAATTAATGAAAAAGAA |
| BL-1F-BC9 | ACACACGCGAGACAGAGAGGCACAAATTAATGAAAAAGAA |
| BL-1F-BC10 | ACGCGCTATCTCAGAGGAGGCACAAATTAATGAAAAAGAA |
| BL-1F-BC11 | CTATACGTATATCTATGAGGCACAAATTAATGAAAAAGAA |
| BL-1F-BC12 | ACACTAGATCGCGTGTGAGGCACAAATTAATGAAAAAGAA |
| BL-1F-BC13 | CTCTCGCATACGCGAGGAGGCACAAATTAATGAAAAAGAA |
| BL-1F-BC14 | CTCACTACGCGCGCGTGAGGCACAAATTAATGAAAAAGAA |
| BL-1F-BC15 | CGCATGACACGTGTGTGAGGCACAAATTAATGAAAAAGAA |
| BL-1F-BC16 | CATAGAGAGATAGTATGAGGCACAAATTAATGAAAAAGAA |
| BL-1F-BC17 | CACACGCGCGCTATATGAGGCACAAATTAATGAAAAAGAA |
| BL-1F-BC18 | TCACGTGCTCACTGTGGAGGCACAAATTAATGAAAAAGAA |
| BL-1F-BC19 | ACACACTCTATCAGATGAGGCACAAATTAATGAAAAAGAA |
| BL-1F-BC20 | CACGACACGACGATGTGAGGCACAAATTAATGAAAAAGAA |
| BL-1F-BC21 | CTATACATAGTGATGTGAGGCACAAATTAATGAAAAAGAA |
| BL-1F-BC22 | CACTCACGTGTGATATGAGGCACAAATTAATGAAAAAGAA |
| BL-1F-BC23 | CAGAGAGATATCTCTGGAGGCACAAATTAATGAAAAAGAA |
| BL-1F-BC24 | CATGTAGAGCAGAGAGGAGGCACAAATTAATGAAAAAGAA |
| BL-1F-BC25 | CGCGACACGCTCGCGCGAGGCACAAATTAATGAAAAAGAA |
| BL-1F-BC26 | CACAGAGACACGCACAGAGGCACAAATTAATGAAAAAGAA |
| BL-1F-BC27 | CTCACACTCTCTCACAGAGGCACAAATTAATGAAAAAGAA |
| BL-1F-BC28 | CTCTGCTCTGACTCTCGAGGCACAAATTAATGAAAAAGAA |
| BL-1F-BC29 | TATATATGTCTATAGAGAGGCACAAATTAATGAAAAAGAA |
| BL-1F-BC30 | TCTCTCTATCGCGCTCGAGGCACAAATTAATGAAAAAGAA |
| BL-1F-BC31 | GATGTCTGAGTGTGTGGAGGCACAAATTAATGAAAAAGAA |
| BL-1F-BC32 | GAGACTAGAGATAGTGGAGGCACAAATTAATGAAAAAGAA |
| BL-1F-BC33 | TCTCGTCGCAGTCTCTGAGGCACAAATTAATGAAAAAGAA |
| BL-1F-BC34 | ATGTGTATATAGATATGAGGCACAAATTAATGAAAAAGAA |
| BL-1F-BC35 | GCGCGCGCACTCTCTGGAGGCACAAATTAATGAAAAAGAA |
| BL-1F-BC36 | GAGACACGTCGCACACGAGGCACAAATTAATGAAAAAGAA |
| BL-1F-BC37 | ACACATATCGCACTACGAGGCACAAATTAATGAAAAAGAA |
| BL-1F-BC38 | GTGTGTCTCGATGCGCGAGGCACAAATTAATGAAAAAGAA |
| BL-1F-BC39 | CGCACACATAGATACAGAGGCACAAATTAATGAAAAAGAA |
| BL-1F-BC40 | TGTCATATGAGAGTGTGAGGCACAAATTAATGAAAAAGAA |
| BL-2R-BC1 | CGCACTCTGATATGTGGACTTTATTTTTCCAGTTACTTTTT |
| BL-2R-BC2 | CTCACAGTCTGTGTGTGACTTTATTTTTCCAGTTACTTTTT |
| BL-2R-BC3 | CTCTCACGAGATGTGTGACTTTATTTTTCCAGTTACTTTTT |
| BL-2R-BC4 | CGCGCGTGTGTGCGTGGACTTTATTTTTCCAGTTACTTTTT |
| BL-2R-BC5 | CACATAGAGATACAGAGACTTTATTTTTCCAGTTACTTTTT |
| BL-2R-BC6 | CGCAGCGCTCGACTGTGACTTTATTTTTCCAGTTACTTTTT |
| BL-2R-BC7 | TCTGTCTCGCGTGTGTGACTTTATTTTTCCAGTTACTTTTT |
| BL-2R-BC8 | CTCTGAGATAGCGCGTGACTTTATTTTTCCAGTTACTTTTT |
| BL-2R-BC9 | TCTGTCTCGCGTGTGTGACTTTATTTTTCCAGTTACTTTTT |
| BL-2R-BC10 | CTCTGAGATAGCGCGTGACTTTATTTTTCCAGTTACTTTTT |
| BL-2R-BC11 | ATAGATATACGTATAGGACTTTATTTTTCCAGTTACTTTTT |
| BL-2R-BC12 | ACACGCGATCTAGTGTGACTTTATTTTTCCAGTTACTTTTT |
| BL-2R-BC13 | CTCGCGTATGCGAGAGGACTTTATTTTTCCAGTTACTTTTT |
| BL-2R-BC14 | ACGCGCGCGTAGTGAGGACTTTATTTTTCCAGTTACTTTTT |
| BL-2R-BC15 | ACACACGTGTCATGCGGACTTTATTTTTCCAGTTACTTTTT |
| BL-2R-BC16 | ATACTATCTCTCTATGGACTTTATTTTTCCAGTTACTTTTT |
| BL-2R-BC17 | ATATAGCGCGCGTGTGGACTTTATTTTTCCAGTTACTTTTT |
| BL-2R-BC18 | CACAGTGAGCACGTGAGACTTTATTTTTCCAGTTACTTTTT |
| BL-2R-BC19 | ATCTGATAGAGTGTGTGACTTTATTTTTCCAGTTACTTTTT |
| BL-2R-BC20 | ACATCGTCGTGTCGTGGACTTTATTTTTCCAGTTACTTTTT |
| BL-2R-BC21 | ACATCACTATGTATAGGACTTTATTTTTCCAGTTACTTTTT |
| BL-2R-BC22 | ATATCACACGTGAGTGGACTTTATTTTTCCAGTTACTTTTT |
| BL-2R-BC23 | CAGAGATATCTCTCTGGACTTTATTTTTCCAGTTACTTTTT |
| BL-2R-BC24 | CTCTCTGCTCTACATGGACTTTATTTTTCCAGTTACTTTTT |
| BL-2R-BC25 | GCGCGAGCGTGTCGCGGACTTTATTTTTCCAGTTACTTTTT |
| BL-2R-BC26 | TGTGCGTGTCTCTGTGGACTTTATTTTTCCAGTTACTTTTT |
| BL-2R-BC27 | TGTGAGAGAGTGTGAGGACTTTATTTTTCCAGTTACTTTTT |
| BL-2R-BC28 | GAGAGTCAGAGCAGAGGACTTTATTTTTCCAGTTACTTTTT |
| BL-2R-BC29 | TCTATAGACATATATAGACTTTATTTTTCCAGTTACTTTTT |
| BL-2R-BC30 | GAGCGCGATAGAGAGAGACTTTATTTTTCCAGTTACTTTTT |
| BL-2R-BC31 | CACACACTCAGACATCGACTTTATTTTTCCAGTTACTTTTT |
| BL-2R-BC32 | CACTATCTCTAGTCTCGACTTTATTTTTCCAGTTACTTTTT |
| BL-2R-BC33 | AGAGACTGCGACGAGAGACTTTATTTTTCCAGTTACTTTTT |
| BL-2R-BC34 | ATATCTATATACACATGACTTTATTTTTCCAGTTACTTTTT |
| BL-2R-BC35 | CAGAGAGTGCGCGCGCGACTTTATTTTTCCAGTTACTTTTT |
| BL-2R-BC36 | GTGTGCGACGTGTCTCGACTTTATTTTTCCAGTTACTTTTT |
| BL-2R-BC37 | GTAGTGCGATATGTGTGACTTTATTTTTCCAGTTACTTTTT |
| BL-2R-BC38 | GCGCATCGAGACACACGACTTTATTTTTCCAGTTACTTTTT |
| BL-2R-BC39 | TGTATCTATGTGTGCGGACTTTATTTTTCCAGTTACTTTTT |
| BL-2R-BC40 | ACACTCTCATATGACAGACTTTATTTTTCCAGTTACTTTTT |

**Table S11.** Genbank accessions of reference *ospC* sequences used during sequence clustering.

| *ospC*  type | Accession | Region (as applicable) |
| --- | --- | --- |
| A | [AE000792](https://www.ncbi.nlm.nih.gov/nuccore/AE000792) | 16903 - 17535 |
| B | [CP001422](https://www.ncbi.nlm.nih.gov/nuccore/CP001422) | 16904 - 17539 |
| C | [CP002316](https://www.ncbi.nlm.nih.gov/nuccore/CP002316) | 16909 - 17541 |
| D | [CP001484](https://www.ncbi.nlm.nih.gov/nuccore/CP001484) | 16911 - 17546 |
| E | [CP002239](https://www.ncbi.nlm.nih.gov/nuccore/CP002239) | 16885 - 17514 |
| F | MH071432 |  |
| G | [CP001375](https://www.ncbi.nlm.nih.gov/nuccore/CP001375) | 16911 - 17543 |
| H | [CP001271](https://www.ncbi.nlm.nih.gov/nuccore/CP001271) | 16904 - 17536 |
| I | [CP001446](https://www.ncbi.nlm.nih.gov/nuccore/CP001446) | 16886 - 17518 |
| J | [CP001535](https://www.ncbi.nlm.nih.gov/nuccore/CP001535) | 16910 - 17545 |
| K | [CP002268](https://www.ncbi.nlm.nih.gov/nuccore/CP002268) | 16902 - 17537 |
| L | X81524 |  |
| M | [CP001550](https://www.ncbi.nlm.nih.gov/nuccore/CP001550) | 16917 - 17555 |
| N | MH071433 |  |
| O | MH071434 |  |
| P | U91796 |  |
| Q | U91790 |  |
| R | U91800 |  |
| S | U91793 |  |
| T | MH071436 |  |
| U | [CP001493](https://www.ncbi.nlm.nih.gov/nuccore/CP001493) | 16908 - 17549 |
| X | HM047876 |  |
| Y | HM047875 |  |
| A3 | EF592541 |  |
| B3 | MH071430 |  |
| C3 | EF592543 |  |
| D3 | EF592544 |  |
| E3 | EF592545 |  |
| F3 | EF592547 |  |
| H3 | FJ932733 |  |
| I3 | FJ932734 |  |
| C14 | MH071431 |  |
